# Reconstructing Hominin Diets with Stable Isotope Analysis of Amino Acids: New Perspectives and Future Directions

**DOI:** 10.1093/biosci/biac028

**Published:** 2022-05-23

**Authors:** Thomas Larsen, Ricardo Fernandes, Yiming V Wang, Patrick Roberts

**Affiliations:** Department of Archaeology, Max Planck Institute for the Science of Human History, Jena, Germany; University of Oxford, Oxford, England, United Kingdom, and with the Faculty of Arts at Masaryk University, Czech Republic; Department of Archaeology, Max Planck Institute for the Science of Human History, Jena, Germany; School of Social Sciences, University of Queensland, in St Lucia, Queensland, Australia

**Keywords:** Isotope fingerprinting, trophic ecology, human nutrition, archeology, paleoecology

## Abstract

Stable isotope analysis of teeth and bones is regularly applied by archeologists and paleoanthropologists seeking to reconstruct diets, ecologies, and environments of past hominin populations. Moving beyond the now prevalent study of stable isotope ratios from bulk materials, researchers are increasingly turning to stable isotope ratios of individual amino acids to obtain more detailed and robust insights into trophic level and resource use. In the present article, we provide a guide on how to best use amino acid stable isotope ratios to determine hominin dietary behaviors and ecologies, past and present. We highlight existing uncertainties of interpretation and the methodological developments required to ensure good practice. In doing so, we hope to make this promising approach more broadly accessible to researchers at a variety of career stages and from a variety of methodological and academic backgrounds who seek to delve into new depths in the study of dietary composition.

Investigating hominin diets and environments  has long been at the forefront of paleoanthropological and archeological research. In deep time contexts, there have been major debates as to the degree to which increasing meat consumption and procurement in more open environments set the hominin clade apart from the lineages of our closest extant great ape relatives (e.g., Domínguez-Rodrigo et al. 2014). Physiologically, the ability to target nutritionally dense foods and process (e.g., through cooking) dietary resources has been linked to hominin trajectories of expanding brains, lengthening of small intestines, and shrinking large intestines (Armelagos 2014). Meanwhile, in the context of our own species’s evolution in Africa approximately 300,000 years ago and expansion around much of the globe by the end of the Late Pleistocene, it has been proposed that *Homo sapiens* demonstrates a dietary flexibility and adaptiveness not seen among other hominins (Roberts and Stewart 2018). Investigations of Holocene humans have been crucial for exploring the potential social, economic, and political ramifications of the emergence of food production (Richards et al. 2003, Jones and Rowley-Conwy 2016) and, later, urbanism (Twiss 2012, Styring et al. 2017) in the archeological record. Certainly, in ethnographic, historical, and archeological contexts, the processing, consumption, and procurement of food have persistently played a major role in cultural expression, political demarcation, economic organization, and adaptations to changing climates and environments (e.g., Forson and Counihan 2013), all issues that remain important topics in a dynamic twenty-first century world. The detailed investigation of hominin nutrition, dietary change, and variabilities in food sources across space and time therefore has much to tell us about where we come from and where we are going.

Much of what we know about past hominin nutrition, physiology, and diet comes from observational studies of modern populations and physical examination of skeletal remains and artifacts from burials and excavation sites (Ungar et al. 2006, Reed 2021). In particular, the analysis of the organic remnants of plants and animals can provide detailed snapshots into what past hominins might have been eating (Hedges 2009), as well as the ecosystems in which they might have lived (Beuning et al. 2011). From the 1970s onward, however, stable isotope analysis of bulk organic and inorganic hominin tissues emerged as an important means of directly providing broad overall perspectives on how much of different food sources individuals might have been consuming (Van Der Merwe and Vogel 1978, Lee-Thorp et al. 1989), often even yielding dietary information in contexts in which the remains of food sources may not themselves be preserved (Pate 1994). The stable isotope ratios of bulk nitrogen (*δ*^15^N) are usually used to infer trophic position as the enrichment of ^15^N of the total proteinaceous tissue is generally 3‰–5‰ over that of the diet (Smith and Epstein 1971). By contrast, the stable isotope ratio of bulk carbon (*δ*^13^C) has been primarily used as an ecological source tracer because ^13^C enrichment during trophic transfer is small, usually less than 1‰, but *δ*^13^C varies predictably among different plant groups (e.g., C_3_ and C_4_) and environmental settings (van der Merwe and Medina 1991). Nevertheless, during the last two decades, it has become increasingly clear that dietary reconstructions based on bulk stable isotope ratios are hindered by two major complicating factors. First, numerous food sources can have similar *δ*^13^C and *δ*^15^N values, limiting the resolution of interpretation. Second, the isotopic baselines (i.e., *δ*^13^C and *δ*^15^N values for food sources at the base of an ecosystem) can vary significantly with location, season, and environment. For example, the *δ*^15^N range between nitrogen-fixing plants and those fertilized by seabird guano can be as large as 20‰ in plant tissues (Szpak et al. 2014), and it is not unusual that the *δ*^13^C range of algal biomass produced during low and high productivity periods is 10‰ (Guiry 2019). In paleodietary studies, it can be particularly challenging to obtain this baseline information and therefore reliably and accurately interpret hominin dietary choices.

The three main determinants of stable isotope ratios in consumer tissues are diet; digestive processes; and, for stable carbon isotopes, the dietary routing of carbohydrates, protein, and lipids to tissue synthesis (Fernandes et al. 2012, Hobbie 2017). Although it may not be feasible in an archeological context, accurate interpretation of bulk stable isotope ratio data from collagen, the main organic constituent in bones, is based on knowledge of the composition and isotopic values of dietary macronutrients. The isotopic values are often estimated from measurements of food remains (e.g., animal bone collagen, charred plants), although the main edible fractions may not be available for analysis. In this case, estimates of the isotopic values of the edible fractions are made from known offsets between the measured fractions in archeological samples (e.g., bone collagen versus muscles; Fernandes et al. 2015, Bownes et al. 2017, Soncin et al. 2021). As for macronutrient food composition, modern composition values are sometimes employed, although these can vary as a result of several factors (e.g., the fat content of consumed flesh from different animal species may depend on catch season and on the selection of cuts and organs for consumption, the effects of cooking). An alternative approach to estimating macronutrient contributions for different food types relies on data for average human consumption patterns (Fernandes et al. 2015). Dietary estimates can be obtained using mixing models that incorporate multiple parameters (food macronutrient content and isotopic values, dietary routing, and consumer-to-diet isotopic offsets; Fernandes et al. 2015, Cheung and Szpak 2020). However, their estimate precision may be limited, given the compounded uncertainties in these parameters.

To overcome food source equifinality, baseline issues, and the uncertainties regarding dietary routing associated with bulk stable isotope ratios, archeologists are increasingly using amino acid stable isotope ratios. These nitrogenous molecules are key for nutrient exchange, because every living organism synthesizes its proteins from the same set of 20 amino acids. They are also used to form other biomolecules or are oxidized to urea and carbon dioxide as a source of energy. Pioneering work three decades ago on both modern and fossil proteins recognized that distinct metabolic processes control isotope patterns among individual amino acids in consumers and their food sources (Edgar Hare et al. 1991). However, the great potential of using these patterns to determine dietary variability only emerged in the following decade from collective evidence gathered across multiple fields. Amino acids can be functionally divided into two groups on the basis of their carbon skeletons (Borman et al. 1946, Womack and Rose 1947, Reeds 2000). The first group, the essential amino acids, cannot be synthesized in the body and must therefore be provided by the organism's diet. The second group, the nonessential amino acids, either can be provided directly by the diet or can be synthesized in the body from metabolic intermediates derived from dietary fat, carbohydrates, and proteins (box 1). Despite the organism's ability to *de novo* synthesize nonessential amino acid carbon skeletons, it is not always achieved at a rate that meets metabolic demand and may become limiting for growth and vital metabolic functions (Womack and Rose 1947, Horvath et al. 1996). A separate set of metabolic processes control the fate of amino acid nitrogen (box 1). Trophic amino acids readily transaminate and exchange nitrogen with other amino acids during trophic transfer in contrast with the source amino acids that largely retain their *δ*^15^N amino acid values. Since these metabolic processes cause a much greater ^15^N enrichment in the trophic than source amino acids, researchers are increasingly using paired isotopic spacing as means of inferring trophic position while circumventing the issue of varying isotopic baselines (McClelland and Montoya 2002, Popp et al. 2007, Yoshito et al. 2007, Ohkouchi et al. 2017).

Box 1. **Metabolism of amino acid nitrogen and carbon follows distinct pathways.**

The schematic representations of amino acid metabolic pathways in figure 1 are depicted in the liver because it is the body's main hub for amino acid metabolism and protein synthesis, degradation and detoxification. Exceptions include the branched chain amino acids (leucine, isoleucine, valine), where the initial catabolic step takes place in extrahepatic tissues (i.e., tissues beyond the liver). Pertaining to nitrogen, the amino nitrogen is transaminated to ammonia. The excess ammonia is converted into urea in the liver through the urea cycle, and the remaining ammonia becomes part of the metabolic amino nitrogen pool. Those amino acids whose nitrogen can be readily exchanged in a single chemical step, the trophic amino acids, most likely share this metabolic amino nitrogen pool with other trophic amino acids (O'Connell 2017). The source amino acids do contribute to the common amino nitrogen via irreversible transamination. An anomaly to the trophic and source amino acids is threonine, which is denoted as a metabolic amino acid, because it is ^15^N depleted during trophic transfer (Edgar Hare et al. 1991).

**Figure 1.**
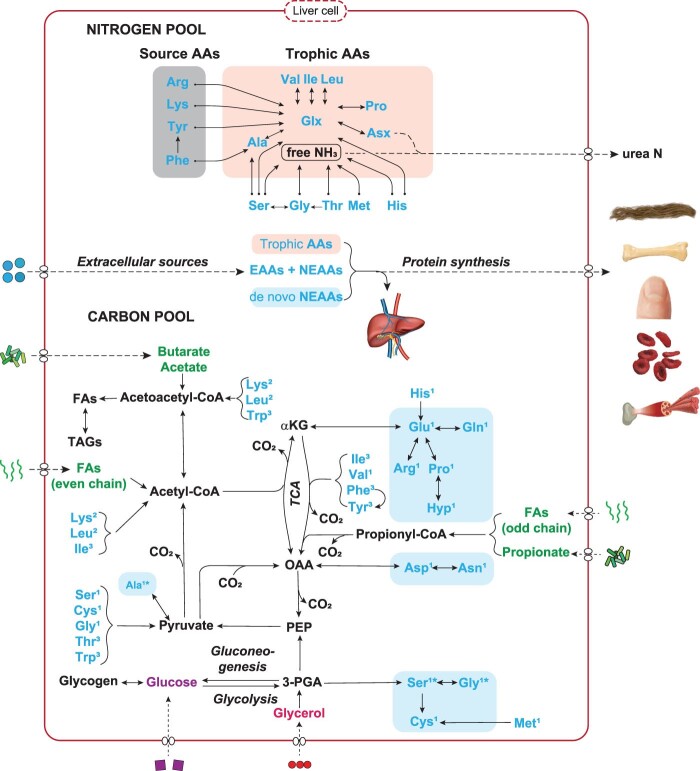
Metabolism of amino acid nitrogen and carbon follows distinct pathways.

Pertaining to the carbon skeletons, the nonessential amino acids can be grouped according to their association with biosynthetic pathways. The glycolytic amino acids are synthesized from metabolic intermediates (3-phosphoglyceric acid, phosphoenolpyruvic acid) of the glycolytic pathway (in the cytosol) and the tricarboxylic acid cycle (α-Ketoglutaric acid, oxaloacetate) amino acids are synthesized from intermediates of the Krebs cycle (in the mitochondria). Glucose and glycerol are sourced to the glycolytic pathway, and fatty acids and short-chain fatty acids are sourced to the tricarboxylic acid cycle. Tricarboxylic acid products can also function as intermediates for Ala via routing to phosphoenolpyruvate and pyruvate. The catabolism of excess amino acids either occurs via gluconeogenesis or ketogenesis. Gluconeogenesis is the synthesis of glucose from noncarbohydrate precursors such as the glucogenic amino acids (marked with a 1) and ketogenesis is the metabolic pathway for producing ketone bodies by breaking down fatty acids and ketogenic amino acids (marked with a 2). A large group of amino acids can be catabolized by both processes (marked with a 3). The broken lines indicate transportation of metabolites across the cell membrane. Images from macrovector (http://freepik.com) under Creative Commons license.

Archeologists, often in collaboration with biochemists and ecologists, have contributed with insights about the controls of amino acid stable isotope ratio patterns via controlled feeding studies and phenomenological studies (Edgar Hare et al. 1991, Fogel and Tuross 1999, 2003, Corr et al. 2005, Honch et al. 2012, Jarman et al. 2017, Webb et al. 2017). Increasingly, amino acid stable isotope ratio analysis has also been applied to varying archeological and paleoecological contexts, from Neanderthal dietary preferences and complexity relative to *Homo sapiens* (Naito et al. 2016a) to the diets of Polynesian populations living on the supposedly ecologically vulnerable island of Rapa Nui (Easter Island; Jarman et al. 2017, Commendador et al. 2019). Although such studies hold much potential and although these methodologies have been well reviewed for ecological (Nielsen et al. 2018, McMahon and Newsome 2019, Whiteman et al. 2019)—and, to a lesser extent, archeological—contexts (O'Connell and Collins 2018), they have often remained something of a black box for the wider archeological and anthropological communities, with the potential and limitations of amino acid stable isotope ratios in reconstructing past hominin diets and ecologies often remaining obscured. Meanwhile, ecologists and biochemists applying methods to archeological contexts might be unfamiliar with the particular taphonomic and interpretive issues of deep time hominin diets. In the present article, we highlight the utility of amino acid stable isotope ratios for deep investigations of hominin diets across time and space and discuss the next steps required for an expanded use of amino acid stable isotope ratios in archeology, paleoanthropology, and paleoecology. We will first outline how human digestive physiology and metabolic pathways affect amino acid stable isotope ratio patterns and then showcase *δ*^15^N_amino acid_ and *δ*^13^C_amino acid_ applications via case studies and compiled literature data. In particular, we seek to demonstrate how amino acid stable isotope ratio patterns remain diagnostic of trophic position and food source, regardless of varying isotope baselines, but also how the potential of integrating baseline information with multivariate amino acid stable isotope ratio analysis can provide more robust insights into food sources. We propose a series of best practices for obtaining reliable amino acid stable isotope ratio results and for the statistical analysis of multivariate isotope tracer data. We also outline a series of perspectives and possibilities relating to this methodology moving forward. In doing so, we seek to make these approaches and their potential and pitfalls in the context of hominin diet and paleoecological reconstruction more accessible to researchers bridging the fields of ecology and anthropology.

### Factors affecting isotope values

Beyond dietary practices, food processing, digestive and metabolic processes can affect amino acid stable isotope ratio values in major ways.

### Food processing

Cooking can be construed as a form of external predigestion that lowers the activity of certain antinutrients and the overall protein quality (Candela et al. 1997, Chau et al. 1997). No recent human populations are known to have lived without cooking, and it likely played a major role in influencing hominin diets and metabolisms from the Pliocene–Pleistocene onward (Wrangham and Conklin-Brittain 2003, Zink and Lieberman 2016). The ability to increase the nutritional value of plant- and animal-derived foods and access difficult to digest nutrients has opened up metabolic energy for other aspects of hominin biology, including brain growth (Aiello and Wheeler 1995), although this remains disputed (Cornélio et al. 2016). This is one reason the advent of cooking and the control of fire in the archeological and paleoanthropological records have received so much attention (Attwell et al. 2015, Smith et al. 2015, Wrangham 2017).

Poorly digestible animal proteins, such as collagen, elastin, and keratin, cannot be digested by enzymes in the stomach and small intestine (Becker and Yu 2013), and many plant macronutrients also resist digestion, especially those in legumes and tubers, unless they are cooked (Zink and Lieberman 2016). Other food-processing techniques, such as fermentation, soaking, and sprouting, are also important for detoxifying starchy plants or removing antinutrients (Capparelli et al. 2011). Food processing may, however, also negatively affect enzymatic digestibility, solubility, and intestinal absorption of certain plant proteins. Proteins may become further polymerized during cooking (Yu et al. 2017), and high heat causes Maillard reactions between sugar and proteins that lead to irreversible protein modifications and a diverse range of indigestible compounds (Hemmler et al. 2018). Although it is well established that cooking modifies the protein, carbohydrate, and lipid contents of foods—and, consequently, bulk stable isotope ratios to some extent (Royer et al. 2017)—more work is required to establish how cooking affects amino acid stable isotope ratios.

### Digestion and metabolic processes

We tend to be isotopically heavier than the food we eat, because digestive and metabolic processes preferentially oxidize isotopically lighter molecules that leave our bodies as carbon dioxide and urea. The first step in this multistep processes is conversion of carbohydrates, fats, and proteins into smaller molecules that can be absorbed by the lining of the small intestines (figure 2). Some nonessential amino acids, such as glutamine, glutamic acid, and aspartic acid, are catabolized extensively for oxidative fuel in the mucous membrane or used as building blocks for synthesizing other nonessential amino acids (Burrin and Stoll 2009). A lack of these nonessential amino acids in the diet can lead to increased catabolism of particular essential amino acids, such as leucine, which means that they become unavailable for the formation of structural tissues. Likewise, nutrient deprivation may increase the oxidation of dietary amino acids in the gut (Neis et al. 2015). Therefore, nonessential amino acids can be viewed as functionally essential if the supply does not meet metabolic demand (Womack and Rose 1947, Horvath et al. 1996).

**Figure 2.**
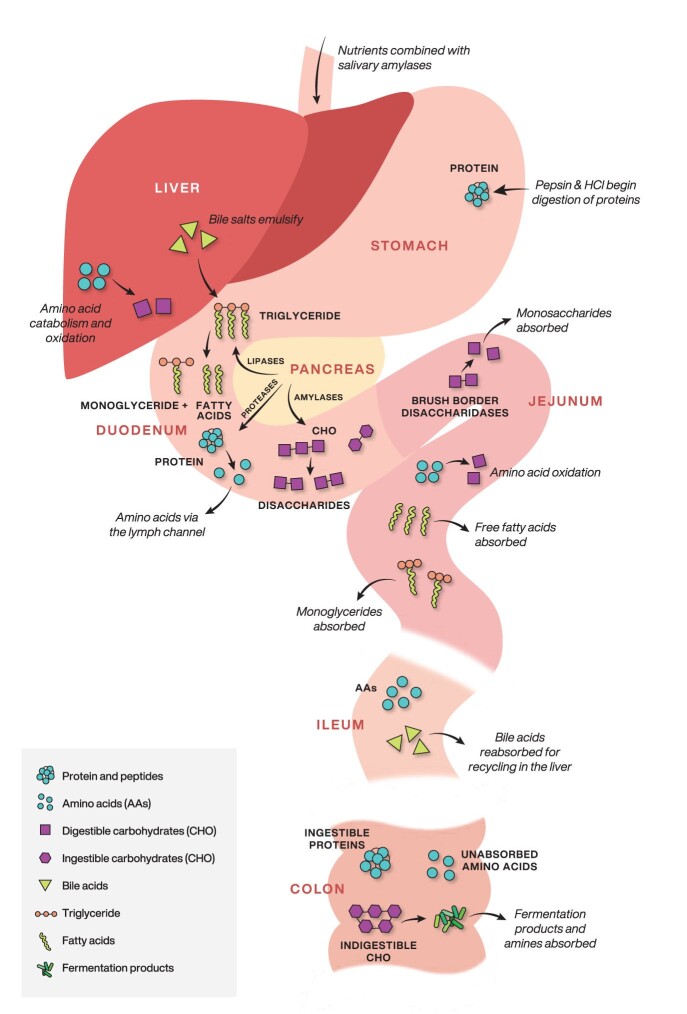
The goal of the digestive processes is to convert food into molecules small enough to be absorbed by the epithelial cells of the intestinal villi. After mastication and mixing with salivary amylases in the mouth, pepsin and HCl begin digestion of proteins in the stomach after which the pancreas releases lipases, proteases and amylases to the small intestine. Bile salts released from the gallbladder help to emulsify fats. Most nutrient absorption takes place in the first and middle parts of the small intestine, the duodenum and jejunum. The last part of the small intestine, the ileum, mostly absorbs vitamin B12, bile salts, and absorbed digestion products that were not absorbed by the jejunum. Absorbed amino acids are transported to the liver or catabolized for oxidative fuel in the mucous membrane of the small intestine. The nutrients that escape primary digestion in the small intestine become available as a substrate for the microbiota in the colon. The most important microbial fermentation by-products from these processes are the short-chain fatty acids, which, on reaching the liver, modulate glucose metabolism and fat deposition and source intermediates for nonessential amino acid synthesis. Source: The figure was modified from Jeejeebhoy (2002).

The nutrients that escape primary digestion in the small intestine become available as a substrate for the microbiota community in the large intestine, the colon (Oliphant and Allen-Vercoe 2019). The primary fermentation products in the colon include various short-chain fatty acids and amine by-products from basic amino acids and phenols or indoles from aromatic amino acids (figure 2). The most important microbial fermentation by-products, the short-chain fatty acids, can be an intermediate for nonessential amino acid synthesis (Oliphant and Allen-Vercoe 2019). There is also evidence that a process is known as *urea nitrogen salvaging* may be responsible for gut microbial contributions of essential amino acids, such as lysine and threonine, to the mammalian host (Metges 2000, Torrallardona et al. 2003). Urea, a highly water-soluble molecule, is the end nitrogenous product of protein catabolism. Once urea is passed via the blood into the gastrointestinal tract, microbes in both the small and large intestines may catabolize it into ammonia or use it as a nitrogen source for amino acid *de novo* synthesis (Stewart and Smith 2005). This process allows the host to salvage nitrogen, mostly in the form of ammonia and, to a much lesser degree, microbially synthesized amino acids. Therefore, a small part of the organism's metabolic demand for essential amino acids may be met by the gut microbes but, as was reviewed by Fuller (2012), the degree to which dietary levels of proteins and complex carbohydrates, residence time of the digesta, and taxonomic assemblage of gut microbes affect essential amino acid supplementation to the host is not well understood (but see a recent rodent study by Newsome et al. 2020).

Digestive and metabolic processes alter amino acid stable isotope ratios. The most commonly used trophic amino acids for estimating trophic position, the *δ*^15^N of glutamic acid and proline, typically increase by approximately 5‰–8‰ per trophic step (McMahon and McCarthy 2016). *δ*^15^N_proline_ values are expected to mirror those of *δ*^15^N_glutamic acid_ because proline can be converted to glutamic acid in two catabolic steps that do not involve transamination (box 1; Fichman et al. 2015). In contrast to the trophic amino acids, the source amino acids cannot readily exchange nitrogen with the metabolic pool (box 1). As a result, commonly used source amino acids, such as phenylalanine and lysine, remain relatively unaltered as they move through the food chain (McMahon and McCarthy 2016). The *δ*^15^N change in source relative to trophic amino acids during trophic transfer is called the *trophic discrimination factor* (TDF). In hominin studies, the most applied TDF value for glutamic acid to phenylalanine (Glx–Phe) spacing is 7.5‰, although the degree to which this value is affected by turnover rates, nutritional demands and the macromolecular composition of the diets remains elusive. A feeding trial with mice showed that higher protein led to smaller TDF_Glx__–__Phe_, probably because proteins were used as an energy source instead of lipids (Whiteman et al. 2021). However, the TDF for proline to leucine spacing (TDF_Pro__–__Lys_) did not change significantly, indicating that these two amino acids may provide a more precise estimate of trophic position when the diet quality is unknown (figure 3). Threonine, which becomes ^15^N depleted during trophic transfer, has been proposed as a new biomarker for protein consumption (Fuller and Petzke 2017), but more studies are needed to understand how δ^15^N_threonine_ is affected by diet quality (Whiteman et al. 2021).

**Figure 3.**
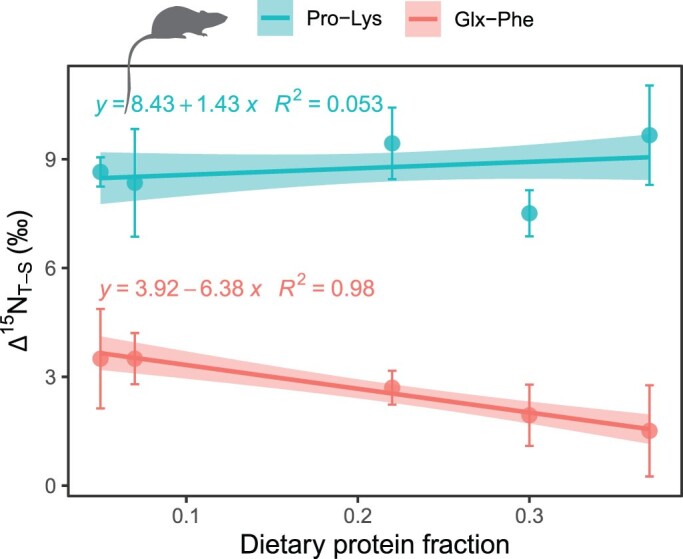
Regressions for pairs of amino acids between dietary protein content and the δ^15^N offset (Δ) between trophic-source amino acids in mice muscle tissue. Higher dietary protein leads to smaller Δ for the glutamic acid–phenylalanine but not the proline–lysine, pair indicating that the latter may provide more precise trophic position estimates in mammalian omnivores when the diet quality is unknown. The shading represents the 95% confidence interval of the regression lines for each trophic-source amino acid pair. Source: The data are from Whiteman and colleagues (2021). Image: Silhouette from PhyloPic (http://phylopic.org) under a Creative Commons license.

Interpreting dietary information from nonessential amino acid *δ*^13^C patterns is complex because of the diverse pathways for producing intermediates used for nonessential amino acid *de novo* synthesis (box 1). Furthermore, in addition to the three main exogenous sources of metabolic intermediates—carbohydrates, fats, and proteins—normal mammalian metabolism also involves the extensive turnover and degradation of endogenous amino acids and lipids. However, controlled feeding trials and epidemiological studies have shown the *δ*^13^C of alanine, glycine, and glutamic acid to be a promising marker of the balance and content of dietary carbohydrates and fat (Choy et al. 2013, Wang et al. 2018, 2019, Yun et al. 2020, Johnson et al. 2021). It is possible to distinguish between these two nutrient fractions because the lipid moieties, glycerol and fatty acids, are ^13^C depleted relative to proteins and carbohydrates owing to isotopic fractionation caused by the enzyme pyruvate dehydrogenase that connects the glycolysis and gluconeogenesis pathways with the tricarboxylic acid cycle (Deniro and Epstein 1977, Melzer and Schmidt 1987, Weber et al. 1997).

Biological variation also poses some challenges for *δ*^13^C_amino acid_ and *δ*^15^N_amino acid_ interpretation. For example, metabolic variations among species, individuals of the same species and even tissues of the same individual have been shown to affect *δ*^15^N_amino acid_ discrimination (McMahon and McCarthy 2016). For *δ*^13^C_amino acid_, a study with pigs fed varying proportions of terrestrial and marine proteins showed that valine, an essential amino acid, tended to be ^13^C depleted in bone collagen and ^13^C enriched in muscle tissue relative to diets (Webb et al. 2017). This finding is significant and reinforces the need to define tissue-specific *δ*^13^C_amino acid_ offsets to reconstruct consumer diets and resource use accurately. Of particular importance to elucidating the degree of dietary, nutritional, and metabolic resolution available from *δ*^13^C_amino acid_ and *δ*^15^N_amino acid_ analyses of archeological and ecological tissues is the design of feeding trial studies that consist of naturally available foods rather than processed foods that bear little comparison to real-world scenarios.

### Isotopic variation of dietary sources

The protein source of consumers will significantly influence the accuracy of estimates of the trophic position of amino acid stable isotope ratios. Based on *δ*^15^N_amino acid_ analysis of cultivated and wild primary producers, Chikaraishi and colleagues (2009, 2010) first identified that the difference between the trophic and source amino acids, also termed the beta (*β*) value, is much lower in terrestrial than aquatic primary producers. However, it is becoming increasingly evident that it is not habitat type (terrestrial versus aquatic), but the degree of vascularization (formation of lignin rich structural tissues) that determines the *β* value in photoautotrophs (Bol et al. 2002, Styring et al. 2014, 2015, Kendall et al. 2019, Takizawa and Chikaraishi 2017, Takizawa et al. 2017). A recent meta-analysis showed the *β*_Glx__–__Phe_ value to be –6.6‰ (standard deviation [SD] = 3.4‰) for vascular autotrophs and 3.3‰ (SD = 1.8‰) for nonvascular autotrophs (Ramirez et al. 2021). The same study also showed that that the *β* values of glutamic acid to leucine (2.5‰, SD = 1.6‰) are considerably less variable than the *β*_Glx__–__Phe_ values in vascular plants. Since vascularization greatly affects trophic position estimates, it is important for paleodietary and paleoecological reconstructions to rely on additional archeological and biogeochemical evidence to define the most realistic *β* values. The following expression can be used to estimate trophic position, provided that both TDF and *β* values are well defined:

**Figure:**
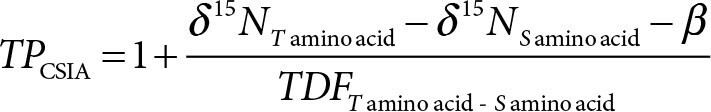


where *T* and *S* signify trophic and source amino acids, respectively. In terms of carbon, essential amino acids are powerful tracers of basal resources in part because *δ*^13^C_essential amino acid_ values in most animals match those in source protein with little or no isotopic offsets, in contrast to the nonessential amino acids where the offsets are typically much greater (McMahon et al. 2010, Barreto-Curiel et al. 2017, Webb et al. 2017, Liu et al. 2018, Wang et al. 2018, 2019, Takizawa et al. 2020, Xu et al. 2021). However, more controlled feeding studies on mammalian model species are needed to validate the extent to which *δ*^13^C_essential amino acid_ values persist through multiple trophic transfers (Webb et al. 2017). Another key feature of the essential amino acids is that the primary organisms synthesizing them—algae, bacteria, fungi, and terrestrial plants—have distinct *δ*^13^C_amino acid_ patterns in which the relative differences among amino acids are consistent, regardless of the actual baseline *δ*^13^C values (Scott et al. 2006, Larsen et al. 2009, Larsen et al. 2013). These patterns are termed fingerprints when they are unique and unequivocal for a given basal resource. These diagnostic *δ*^13^C_essential amino acid_ fingerprints are well suited for retrospective analyses because they remain largely invariant across biogeochemical conditions and trophic transfer (Larsen et al. 2013, 2015, Lynch et al. 2016; Elliott Smith et al. 2018). For example, the range in *δ*^13^C values of algae grown under varying biogeochemical conditions can be as large as 12‰, but mean centering the *δ*^13^C_amino acid_ data can reduce the *δ*^13^C variability among individual essential amino acids by a factor of 10, which makes the fingerprinting approach a much more robust source tracer than bulk stable isotope ratios across time and space (Larsen et al. 2013, 2015). Mean centering is a technique to factor out baseline variability by subtracting the *δ*^13^C mean of all the essential amino acids from the *δ*^13^C of each of the individual essential amino acids. To accurately predict dietary sources with the fingerprinting approach, it is essential to obtain *δ*^13^C_amino acid_ data for the relevant food sources. The general term for the data used to create the classification model is *trainingdata*. Examples of where more training data are needed for pinpointing particular hominin food sources in different contexts include plant organs, such as seeds, nuts, tubers and roots, and plants grown under different abiotic conditions (Paolini et al. 2015, Larsen et al. 2016, Jarman et al. 2017, Bontempo et al. 2020). Likewise, there are too little data available on secondary animal products, such as dairy, and the effects of food preparation on *δ*^13^C_amino acid_ patterns.

## Statistical analyses

Given that amino acid stable isotope ratios involves *δ*^13^C and *δ*^15^N of a variety of amino acids, the resulting number of data points is of a magnitude order higher than those obtained from bulk stable isotope ratio analyses. For this reason, it is important to apply linear transformation techniques to identify the most important features in multivariate amino acid stable isotope ratio data sets, as is the case with the *δ*^13^C_amino acid_ fingerprinting approach mentioned above. Principal component analysis (PCA) is often the choice for exploring *δ*^13^C_amino acid_ variability and patterns in a data set because it is an unsupervised technique (i.e., the data are not grouped *a priori*) that seeks to maximize variability among samples while reducing the number of dimensions. Linear discriminant function analysis (LDA) is a supervised technique that seeks to maximize variability among the predefined groups or classes with the goal of predicting specific protein sources. The number of samples in each linear discriminant group should supersede the number of *δ*^13^C_amino acid_ variables, and the number of samples representing a food source should be greater than the number of essential amino acids. Overall, many amino acid variables as possible are desired to maximize dietary information. An important distinction between PCA and LDA is that only the latter technique assesses the variability of each *δ*^13^C_amino acid_ variable relative to the group mean. To achieve the same for PCA, it is necessary to factor out *δ*^13^C baseline variability by mean centering the data.

A nonmultivariate approach based on paired *δ*^13^C_amino acid_ offsets can also differentiate consumers and predict major protein sources, but it has less predictive power than PCA and LDA, because it relies on three to four amino acids only. Archeologists have traditionally used paired amino acid offsets in conjunction with *δ*^13^C bulk or *δ*^13^C_amino acid_ baselines to identify consumption of high freshwater, marine, terrestrial C_3_, and terrestrial C_4_ protein sources. The offset between glycine and phenylalanine has been used to separate the high terrestrial C_3_ and high freshwater protein groups from the high terrestrial C_4_ and high marine protein groups, and the offset between valine and phenylalanine has been used to separate aquatic and terrestrial resources (Corr et al. 2005, Honch et al. 2012). In principle, it is possible to integrate paired *δ*^13^C_amino acid_ offsets with *δ*^13^C_amino acid_, *δ*^15^N_amino acid_ and even bulk stable isotope ratio values in multivariate models, but only if these variables are completely independent of one another. For example, the amino acids used in paired *δ*^13^C_amino acid_ offsets must be omitted as single amino acid stable isotope ratio variables, and combining both bulk stable isotope ratios and amino acid stable isotope ratio variables for the same element is likewise problematic.

Interpretations of *δ*^13^C_amino acid_ and *δ*^15^N_amino acid_ data in ecological and archeological contexts can also be improved through combination with other isotopic proxies, such as *δ*^34^S_collagen_ or ^14^C_collagen_, in Bayesian mixing models (Fernandes et al. 2014). In the context of paleoanthropology and archeology, the additional application of magnesium and zinc isotopes to trophic level exploration is also opening further proxy possibilities in this regard (Martin et al. 2015, Jaouen et al. 2016). The inclusion of other isotopic proxies must include the biosynthetic pathways associated with each proxy, however see (Fernandes et al. 2015). For instance, bone collagen nitrogen is sourced almost exclusively from dietary protein, whereas collagen carbon is sourced from a mixture of dietary protein, carbohydrate, and fat (Fernandes et al. 2012). It is also important to consider the research question being addressed with a mixing model. This is particularly the case when using concentration independent and dependent models. Concentration independent models employing *δ*^13^C_amino acid_ data estimate the relative proportions of the amino acids contributed from each food source. In contrast, concentration dependent models may estimate the relative mass contribution or relative protein contribution from each source (box 2). When done appropriately, complex Bayesian models can convert amino acid isotopic data into estimations of carbohydrate, protein, and fat sourcing for populations, something previously inconceivable (Fernandes et al. 2014, Larsen et al. 2018, Wyatt et al. 2019).

Box 2. **Schematic illustration of three different mixing models using *δ*^13^C values of essential amino acids as dietary tracers of fish, maize, and leafy vegetables and nuts.**

**Figure 4.**
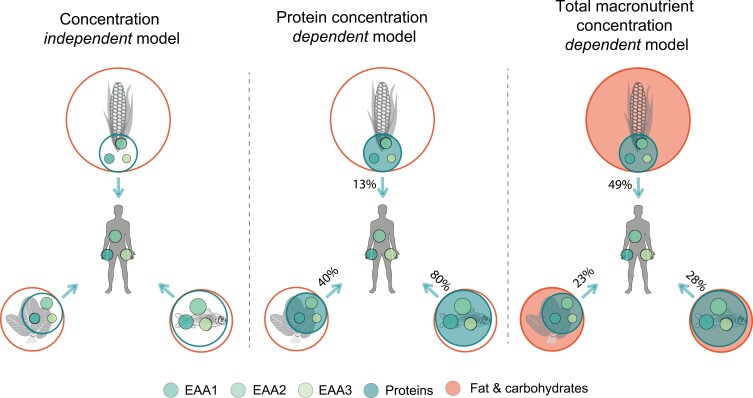
Schematic illustration of three different mixing models using δ^13^C values of essential amino acids as dietary tracers of fish, maize, and leafy vegetables and nuts.

The inner circles in figure 4 represent the weight or caloric values of essential amino acids and proteins relative to the total weight or calories of each food, and the outer circles the relative contribution of each food source. In this example for caloric estimates, the proportion of calories derived from proteins in each food source is 80% for fish, 13% for maize, and 40% for nuts and vegetables, and the relative caloric contribution of each food source is 28% from fish, 49% from maize, and 23% from nuts and vegetables. For simplicity, the essential amino acid proportions relative to proteins are assumed in the present example to be similar in each food source. The concentration independent model quantifies the relative essential amino acid contribution from each food source and carries the assumption that the relative essential amino acid proportions are similar in each food source. The protein concentration dependent model estimates the relative protein contribution from each food source by considering the essential amino acid relative to protein-only calories within each food source. The total macronutrient concentration dependent model estimates the relative caloric contribution of each food source by considering essential amino acid calories relative to total calories. It is important to note that the assimilation efficiency of macronutrients will not necessarily be 100% and may depend on other factors, such as cooking, digestibility, and antinutrients. Thus, effective concentration values that account for such aspects have to be employed in modeling. Some Bayesian software are capable of providing these separate estimates simultaneously when concentration values for essential amino acids and proteins are provided (Fernandes et al. 2012).

## Analytical considerations

Researchers use either gas chromatography–combustion–isotope ratio mass spectrometry (GC–C–IRMS) or liquid chromatography–combustion–isotope ratio mass spectrometry (LC–C–IRMS) to determine amino acid stable isotope ratios. These methods each come with analytical advantages and drawbacks. Of the two systems, LC–C–IRMS produces the most reliable data, but it is only suited for carbon isotope analysis, the required sample amounts are very high, and some essential amino acid peaks cannot be baseline separated (Dunn et al. 2011, Smith et al. 2009). GC–C–IRMS can be used to determine both *δ*^15^N and *δ*^13^C values and the run times are much faster than with the LC–C–IRMS, meaning that it is increasingly the methodology of choice for *δ*^13^C_amino acid_ and *δ*^15^N_amino acid_ in archeological and anthropological contexts. However, the pre- and postanalytical steps are more cumbersome and complex.

The most demanding preanalytical step for preparing samples for GC–C–IRMS analysis is amino acid derivatization. This conversion of polar or nonvolatile compounds into relatively nonpolar or volatile products is usually done by esterifying the carboxylic acid group with an acidified alcohol and acylating the amine, hydroxyl, and thiol groups (Corr et al. 2007b). Derivatization induces kinetic isotope effects and adds additional carbon atoms to the amino acids, and the only way to factor out these effects is by derivatizing a mixture of amino acids with known *δ*^13^C values (Docherty et al. 2001). To minimize the error propagations, it is important to use a surplus of reagents, select reagents and amino acid references with *δ*^13^C values approximating those of the samples of interest, and use a derivatization method that adds as few carbon atoms as possible (e.g., use short-chain alcohols for esterification; Corr et al. 2007a). Although derivatization does not add exogenous nitrogen, there may still be a kinetic isotope effect because of differing rates of chemical reaction of isotopically heavy and light atoms within a given molecule (i.e., isotopologues). This effect can be assessed by comparing the *δ*^15^N values of amino acid standards before and after derivatization (Whiteman et al. 2018).

The accuracy and precision of the isotopic analyses depend on the quality of gas chromatography separation, interface design, and isotopic calibration, which has been discussed in several papers and books (van Leeuwen et al. 2014, Jochmann and Schmidt 2015, Meier-Augenstein 2018). During runs, it is crucial to monitor the isotopic drift of analytical standards and reference materials, such as modern bones, and to ensure that scale normalization for the samples being measured is based on two or more reference analytes (Paul et al. 2007). A GC–C–IRMS system can be kept in good operating condition by monitoring for leakages, inspecting the combustion reactors, regularly changing inlet liners, and shortening or replacing gas chromatography columns. Similarly, it is important to check the relevant interfaces on LC–C–IRMS systems.

The most important proteinaceous tissues in an archeological or anthropological context are skin, hair, nails, ligaments, bones, and teeth. In the archeological record, researchers must often focus on the best-preserved tissue fragments for destructive isotope analysis. Depending on the age of the sample, these fragments can provide insights into the diet and nutrition of a particular life stage. Tissues such as bones, ligaments, and skin are remodeled throughout life but at different turnover rates according to the element in question and the age, sex, and physiological and pathological conditions of the individual (Hadjidakis and Androulakis 2007). In terms of bones, because ribs are remodeled at a much faster rate than femurs, the former will represent a more recent dietary history prior to death than the latter (Tieszen et al. 1983, Fahy et al. 2017). In contrast, tooth dentin and keratin excrescences, such as hair and nails, are not remodeled after formation. Dentin is therefore useful for exploring diets during childhood and adolescence (Sandberg et al. 2014), and hair and nails can inform on diets in the months before death (O'Connell et al. 2001).

With regards to isotopic fractionation, feeding trials with pigs have shown that diet to collagen *δ*^13^C_essential amino acid_ offsets (Δ_t__issue__–d__iet_) can fall outside the 1‰ analytical uncertainty range. Notably, Δ_t__issue__–d__iet_ for valine ranged between 1.3‰ and –2.1‰ (Edgar Hare et al. 1991, Webb et al. 2017). We rule out the possibility that memory effects caused the higher than expected Δ_t__issue__–d__iet_ values because the experimental diets were fed to successive generations of pigs. Although isotopic discrimination during digestion and metabolic routing or microbial supplementation of essential amino acids may affect Δ_t__issue__–d__iet_ values, the most parsimonious explanation is probably sample treatment biases. For example, acid hydrolysis of collagen proteins can, in some cases, lead to Δ_t__issue__–d__iet_ values of 2‰–3‰ if the amino acid recovery rates are low (Jim et al. 2003). It is also worth noting from other animal feeding trials that Δ_t__issue__–d__iet_ values for the essential amino acids usually center around 0‰, and the corresponding values for the nonessential amino acids are much greater (McMahon et al. 2010, Barreto-Curiel et al. 2017, Webb et al. 2017, Wang et al. 2019).

Whether archeological tissues faithfully record diet and nutrition through amino acid stable isotope ratios will also depend on diagenetic biases. In terms of preservation, bone structures are sensitive to environmental fluctuations, such as humidity and temperature shifts, because they accelerate amino acid degradation by creating micro fissures and porous structures in biomineralized tissues (Grupe 1995, Maurer et al. 2014). Degradation and residues from conservation treatments can also introduce exogenous materials to tissues, but this is less of an issue for amino acid stable isotope ratios than for bulk stable isotope ratios because it is possible to extract and isolate amino acids bound in tissue proteins. According to a wool degradation study, soil microbial degradation of keratin appears not to be protein selective, which means that *δ*^13^C_amino acid_ and *δ*^15^N_amino acid_ values do not change significantly with degradation (von Holstein et al. 2014). Besides microbial degradation, the protein concentrations decrease with weathering and fossilization (Rapp Py-Daniel 2014). Therefore, the oldest bones suitable for stable isotope ratios of collagen are often found in cool environments where there was a relatively rapid burial, such as in the Denisova Cave in the Siberian Altai, where the oldest hominin collagen containing bone has been estimated to date to 195,000 years ago (Douka et al. 2019). For bones, collagen yields (more than 1%) and quality indices such as atomic carbon to nitrogen ratios (2.9–3.5) and percentage nitrogen content (more than 0.5%) are the most important preservation criteria (Ambrose 1990, Brock et al. 2012). More recently, paleoproteomics has also been showing great promise for evaluating protein quality, because this technique can more accurately evaluate whether poorly preserved samples are suited for amino acid stable isotope ratio analysis than carbon to nitrogen ratio and other preservation quality indicators (Cleland et al. 2021).

It is well documented that the selection of pretreatment methodology can affect the final *δ*^13^C and *δ*^15^N values of measured bone collagen (Pestle et al. 2014) and food sources (Strait and Spalding 2021). In terms of pretreatment biases of collagen samples for amino acid stable isotope ratios, the isotopic relationships between proline and hydroxyproline have been proposed as a data quality marker (O'Connell and Collins 2018, Roberts et al. 2018). Since hydroxyproline is synthesized exclusively from proline after the formation of immature collagen, the *δ*^13^C and *δ*^15^N values of hydroxyproline should match those of proline. Empirical evidence from archeological studies of mammal bone collagen has confirmed a nearly perfect one-to-one relationship between the two nonessential amino acids (O'Connell and Collins 2018, Roberts et al. 2018). However, this one-to-one relationship is only a guide and not a strict criterion for quality control because an analytical issue, such as coelution of one of the amino acids, does not necessarily render the remaining amino acid data invalid. Moreover, more needs to be understood in terms of the catabolic process and associated isotopic fractionations of hydroxyproline and proline during collagen turnover. Another quality control parameter of amino acid stable isotope ratio values in collagen is based on mass balance equations where the relative contributions of each amino acid to collagen isotope values are compared with bulk collagen isotope values (Soncin et al. 2021). Since the amino acids measured by GC–IRMS typically represent 80%–90% of total carbon and nitrogen by mass, amino acid stable isotope ratio values representing total carbon and nitrogen can be made comparable to bulk stable isotope ratio values by factoring in the composition and the number of carbon and nitrogen atoms in collagen (which can be calculated with the ProtParam tool; Gasteiger et al. 2005). An offset between the estimated (amino acid stable isotope ratios) and measured (bulk stable isotope ratios) greater than two standard deviations from the average would be grounds to discard a sample.

## Applications in archeology and paleoecology

It should be clear from the above that, with robust analytical protocols in place, *δ*^13^C_amino acid_ and *δ*^15^N_amino acid_ measurement of hominin tissues has great potential for studying dietary adaptations and resource sourcing of one of the most prevalent terrestrial omnivores on the planet, humans (as well as their hominin ancestors). Although bulk bone collagen *δ*^13^C and *δ*^15^N analyses have been a staple of paleodietary investigations for four decades (Van Der Merwe and Vogel 1978, Krueger and Sullivan 1984, DeNiro 1985, Walker and Deniro 1986), they can have limited interpretive power because of a high ratio of food sources relative to isotope tracers and confounding bulk isotope values among resource groups (e.g., C_4_ and marine resources; Fry 2013). By comparison, *δ*^13^C_amino acid_ fingerprints have a much higher source specificity and, similar to the *δ*^15^N_amino acid_ offsets used for inferring trophic position, they remain comparatively invariable across different environments (Larsen et al. 2013, 2015, Lynch et al. 2016, Elliott Smith et al. 2018). Therefore, *δ*^13^C_amino acid_ and *δ*^15^N_amino acid_ signals of ecological and archeological tissues are providing a clear improvement in the way we biochemically approach the study of past diets and ecosystems. Analyses of these tissues also play a role in improving the inferences that can be drawn from amino acid stable isotope ratio data in other disciplines. Under the compliance of strict ethical guidelines, the large number of human remains from known contexts that have been made available for studies with bulk stable isotope ratios or other biochemical approaches constitute a wealth of potential amino acid stable isotope ratio data that, in terms of quality, resolution, and distribution across space and time, is rarely attainable for other species.

**Figure 5.**
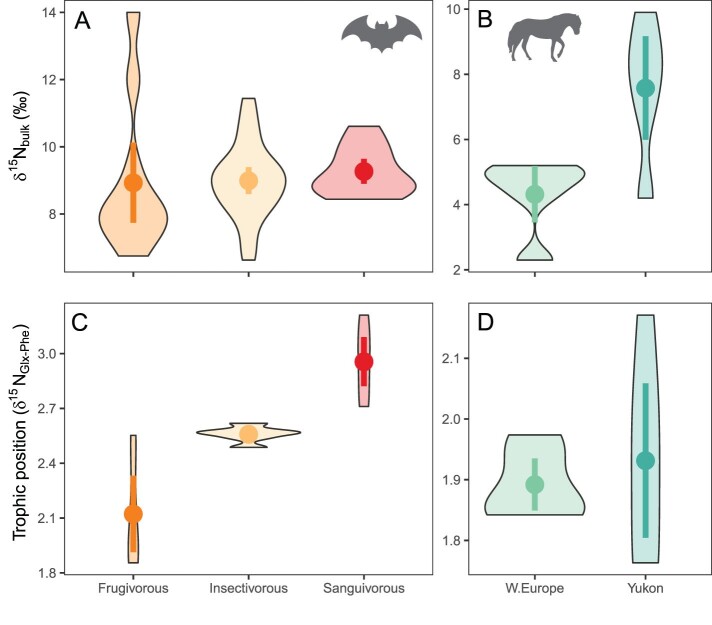
Two case studies of trophic position estimates based on bulk and amino acid Δ^15^N measurements. (a) Bulk δ^15^N analysis of modern frugivorous, insectivorous, and sanguinivorous bat tissues is confounded by environmental variation with the result that the trophic positions of the three species appear to be the same (ANOVA, ***F***(***2,39***) = 0.17, p = .84). (b) δ^15^N_Glx–Phe_ reveals trophic positions congruent with the bats’ known feeding habits (ANOVA, ***F(2,15***) = 22.5, p < .001). (c) In contrast to the modern bat samples, bulk δ^15^N values of Pleistocene horse bone collagen in Western Europe and Yukon are different although they consumed plant foods in both regions (***t***(***6.2***) = –2.96, p < .05). (d) Trophic position based on δ^15^N_Glx–Phe_ spacing of the same samples show that they had the same trophic position in both regions (***t(4.9***) = –0.48, p = .65). We used a TDF of 8.4 and a ***β*** value of –7.6. Source: The data are from Campbell and colleagues (2017) and Schwartz-Narbonne and colleagues (2015). See supplemental appendix S2 for sample information. Image: Silhouettes from PhyloPic (http://phylopic.org) under a Creative Commons license.

The ability of *δ*^15^N_amino acid_ values to overcome the issue of variable *δ*^15^N baselines is best illustrated through studies of nonomnivores fauna. To that end, we compiled bulk and *δ*^15^N_amino acid_ data of modern frugivorous, insectivorous, and sanguinivorous bat tissues (Campbell et al. 2017). Please find the statistical methods for this article in supplemental appendix S1. Despite the different trophic positions of the bats, there is a lack of significant differences in bulk *δ*^15^N values among the three groups due to environmental variations (figure 5a). By contrast, trophic position estimates based on *δ*^15^N_Glx__–__Phe_ values clearly reveal that the three groups are different and accord with observations of their foraging ecology (figure 5c). In a different case study, bulk *δ*^15^N values of Pleistocene horse bone collagen in Western Europe and Yukon are suggestive of a trophic distinction between horses inhabiting these regions (figure 5b; Schwartz-Narbonne et al. 2015). However, *δ*^15^N_Glx__–__Phe_ analysis of the same samples demonstrates this to be a product of baseline *δ*^15^N variation, with equids actually, and, unsurprisingly, inhabiting the same trophic position, in both parts of the world (figure 5d).

Despite the more complex nature of inferring trophic position from *δ*^15^N_Glx__–__Phe_ in omnivores, this proxy has been used successfully to provide detailed dietary information of humans and their hominin relatives. For example, Richards and Trinkaus (2009) concluded, on the basis of both archeological and bulk isotopic data, that Neanderthal diets were heavily meat based, without detectable fish or plant inputs. However, bulk *δ*^15^N methods lack source specificity and may be influenced by regional environmental *δ*^15^N variations (Wang et al. 2014, Styring et al. 2016). Recent baseline-independent *δ*^15^N_amino acid_ analyses of bone collagen from Neanderthals and associated carnivores (e.g., hyaenas and wolves) and herbivores (e.g., mammoths, equids, and deer) have largely confirmed Neanderthals as high trophic level carnivores at sites such as Les Cottés and the Grotte du Renne, in France. However, they have also highlighted dietary contributions of plants in locations such as Spy Cave in Belgium (Naito et al. 2016b, Jaouen et al. 2019). Despite this improved resolution, we still know little about the dietary versatility of Neanderthals, their ability to adapt their foraging strategies to local conditions, and whether their resource gathering strategies were distinct from our own species. Systematic *δ*^15^N_amino acid_ work comparing Neanderthals and *Homo sapiens* in different regions, as well as analysis of Neanderthals across their ever expanding known geographical range, promises to provide further important insights into the dietary adaptability of this hominin group in the future (Belmaker and Hovers 2011, Hallin et al. 2012, Henry et al. 2017).

Although *δ*^15^N_amino acid_ is emerging as an indispensable method for dietary reconstruction, it has limited ability to independently determine proportional contributions of terrestrial versus aquatic resources (i.e., the correct *β* values), which, in turn, can lead to erroneous estimations of trophic positions (see figure 5). This limitation can be reduced in hominin dietary studies by measuring the local δ^15^N values of freshwater fish, terrestrial plants, terrestrial meat, and seafood (Naito et al. 2016a, Drucker et al. 2017, Itahashi et al. 2019). For example, in a *δ*^15^N_amino acid_ study of bone remains from hunter–gatherer and Neolithic populations in the upper Tigris region in the Near East, Itahashi and colleagues (2019) used faunal remains to show that *δ*^15^N_phenylalanine_ values of freshwater fish are lower than those of terrestrial herbivores and carnivores. By employing a trophic position model assuming terrestrial protein contributions, Itahashi and colleagues (2019) inferred high aquatic protein consumption when human bone collagen had a combination of low *δ*^15^N_phenylalanine_ and high trophic position values (Itahashi et al. 2019). Although such approaches rest on a number of assumptions, in relation to both the bulk *δ*^13^C and *δ*^15^N values of faunal remains and the relative abundance of faunal taxa in each excavation layer, the study demonstrated how *δ*^15^N_amino acid_ can help to shed light on resource use—in this case, how a shift from hunting and gathering to farming decreased human dependence on aquatic resources.

**Figure 6.**
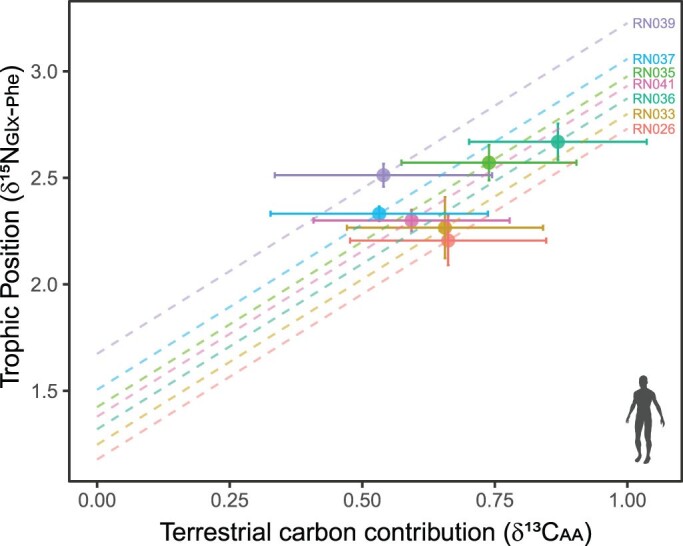
Precolonial Rapa Nui islanders relied on both marine and terrestrial food sources for their sustenance. Therefore, to estimate their trophic positions based on δ^15^N_amino acid_ data from bone collagen a priori knowledge of the proportional dietary contributions of marine versus terrestrial food sources is required. These contributions are depicted along the ***x***-axis. They were estimated with a concentration dependent mixing model (Commendador et al. 2019) based on δ^13^C_essential amino acid_ fingerprinting data (Jarman et al. 2017). The linear isoclines (broken lines) represent the possible trophic position range (fully marine to fully terrestrial) calculated from the δ^15^N_Glx–Phe_ offset using a TDF of 8.4 and β values of –7.6 and 3.3 for terrestrial- and marine-derived food sources, respectively. The trophic position is where the terrestrial carbon contributions meet the isocline. The error bars represent standard deviations. See supplemental appendix S2 for sample information. Image: Silhouettes from PhyloPic (http://phylopic.org) under a Creative Commons license.

The issue of estimating the *β* values of hominins living off of mixed terrestrial and aquatic diets can also be addressed by using *δ*^13^C_essential amino acid_ fingerprints, as exemplified by a joint *δ*^15^N_amino acid_ and *δ*^13^C_amino acid_ data set from prehistoric communities on Rapa Nui (Easter Island) and their potential food sources represented by a mixture of archeological and modern rat, chicken, plant food, and different marine resources (Jarman et al. 2017). The *δ*^13^C_amino acid_ fingerprints and the accompanying concentration-independent mixing model (representing dietary essential amino acids) estimated that the islanders relied much more on marine dietary resources than was previously thought, highlighting the finely tuned environmental adaptations and social resilience of humans on an island previously simplistically discussed as a classic case study of human overexploitation and societal collapse (Diamond 2005). In terms of estimating the islanders’ trophic position, Jarman and colleagues (2017) compared three different dietary scenarios: *β* values based on fully marine, half marine half terrestrial diets, and fully terrestrial diets. Only the 50–50 scenario produced somewhat realistic trophic positions, albeit a quarter of the individuals had trophic positions below 2. This low range can be attributed to an overestimation of marine resources. Therefore, to generate realistic *β* values, we use the estimates produced by Commendador and colleagues (2019) for marine and terrestrial caloric contributions with a concentration-dependent (representing dietary carbon) rather than with a concentration-independent mixing model (figure 6; Jarman et al. 2017). Despite some uncertainty in the proportions of marine to terrestrial contributions, the revised *β* values result in more realistic trophic position estimates ranging between 2.1 and 2.6 (figure 6).

Acknowledging the nuances in routing and metabolic processes and how different *δ*^13^C_amino acid_ and *δ*^15^N_amino acid_ proxies can track these processes, amino acid stable isotope ratio analyses offer opportunities for archeological and paleoecological researchers despite the difficulties presented above. There are remarkably few studies that combine the power of *δ*^13^C_amino acid_ and *δ*^15^N_amino acid_ analyses on the same samples to simultaneously reveal more high-resolution insights into diet, environmental impacts, and trophic relationships. Four recent studies from widely different archeological settings—Neanderthals in France (Naito et al. 2016a, Jaouen et al. 2019), prehistoric humans on Rapa Nui (Jarman et al. 2017), and Romans from Herculaneum—combined *δ*^13^C_amino acid_ and *δ*^15^N_amino acid_ analyses to obtain more robust evidence of dietary patterns (Soncin et al. 2021). The Neanderthal studies applied *δ*^13^C_amino acid_ in conjunction with zooarcheological evidence to rule out the possibility that food processing and consumption of fish and young mammals contributed to unusually elevated collagen *δ*^15^N values, thereby supporting that Neanderthals were indeed top-level carnivores. The Rapa Nui study combined *δ*^13^C_amino acid_ and *δ*^15^N_amino acid_ data to show that islanders were more versatile in their foraging strategies than previously assumed. Finally, the Herculaneum study relied on abundant food remains to make a direct comparison between human bone collagen and food isotopic values for the essential amino acids and source-trophic amino acids to increase the precision of Bayesian dietary estimates.

**Figure 7.**
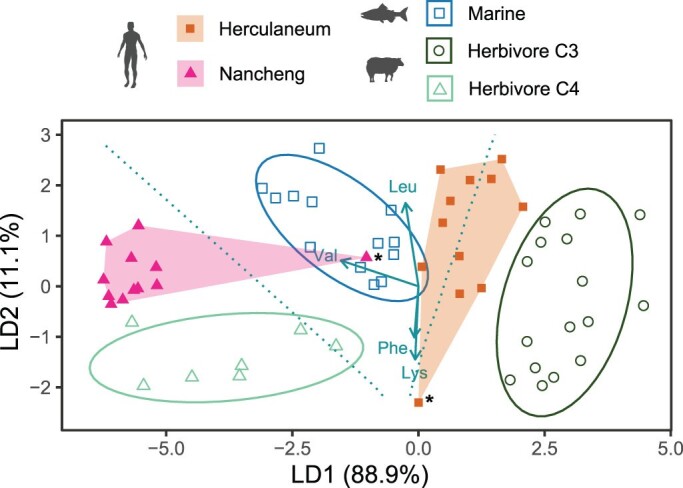
Linear discriminant analysis of compiled δ^13^C_essential amino acid_ data from human and faunal archeological bone collagen based on four essential amino acids (leucine, valine, phenylalanine, and lysine). The training data represent the three faunal groups and the predicted data of the two human sites. The median discriminant values of three faunal groups were significantly different (Pillai's trace = 1.39, ***F(4,66***) = 37.5, p < .001). The ellipses represent 95% prediction intervals of each faunal group, the convex hulls represent the outer group boundaries for each human site, and the arrows represent the relative weightings of the independent variables for creating the discriminant function. The symbols marked with asterisks denote outlier individuals. References: Terrestrial C_3_ (Choy et al. 2010); marine, Herculaneum (Rome, 79 CE; Soncin et al. 2021); and terrestrial C_4_, Nancheng (China, 2000–1600 BCE; Ma et al. 2021). See supplemental appendix S2 for sample information. Image: Silhouettes from PhyloPic (http://phylopic.org) under a Creative Commons license.

A cornerstone for inferring past human diets is analyzing food sources that accurately represent past hominin diets. As was mentioned earlier, the breadth of archeologically relevant training data is still limited. Another constraint is the relatively low number of essential amino acid variables reported in archeological studies to date. Despite these limitations, much can be gleaned from already published *δ*^13^C_amino acid_ data (Ma et al. 2021). To illustrate this, we compiled *δ*^13^C_essential amino acid_ data (leucine, lysine, phenylalanine, and valine) from archeological studies using fauna as proxies for marine, terrestrial C_3_, and terrestrial C_4_ protein sources, and two human populations from C_3_- and C_4_-dominated biomes, respectively. One population was from Herculaneum (Rome, 79 CE) and is documented as living on a diet composed primarily of animal–plant terrestrial C_3_ foods and, to a lesser extent, marine fish (Soncin et al. 2021). The other population from Nancheng (China, 2000–1600 BCE) was argued to be reliant on millet and fauna that foraged on mixed C_3_ or C_4_ vegetation (Ma et al. 2021). The LDA based prediction of human diets largely accord with expectations (figure 7). Most of the Herculaneum individuals cluster with the herbivorous C_3_ fauna indicating that most of their proteins derived from terrestrial sources. The remaining Herculaneum individuals, with one exception (marked with an asterisk), are skewed toward marine protein sources, which may reflect that fish protein contribution is much greater than overall fish caloric contributions (estimated to approximately 10% by Soncin et al. 2021). This is plausible given that proteins in marine fishes represent approximately 80% of their total calories, against 12% in ancient cereals (Liaset and Espe 2008, Boukid et al. 2018). However, we cannot exclude the possibility that tissue to diet isotopic offsets, Δ_t__issue__–d__iet_, shifted LD scores toward marine sources. All the Nancheng individuals except one (marked with an asterisk) plot with the herbivore C_4_ faunal group, but with an offset along LD2 owing to the aforementioned Δ_t__issue__–__d__iet_ or the fact that nonfaunal protein sources, such as millet grains, have slightly different *δ*^13^C_essential amino acid_ fingerprints than the C_4_ grasses eaten by the herbivorous fauna. It is also noteworthy that the elongated ellipse of the C_4_ fauna group along LD1 indicates that some specimens relied more on C_3_ plants than others. The LDA cannot adequately explain the diets of the two outliers: It is unlikely that the Nancheng individual predominantly relied on marine proteins given the site's distance from the ocean, and *δ*^13^C_amino acid_ baseline values of the Herculaneum individual bins it with the other individuals from the site.

**Figure 8.**
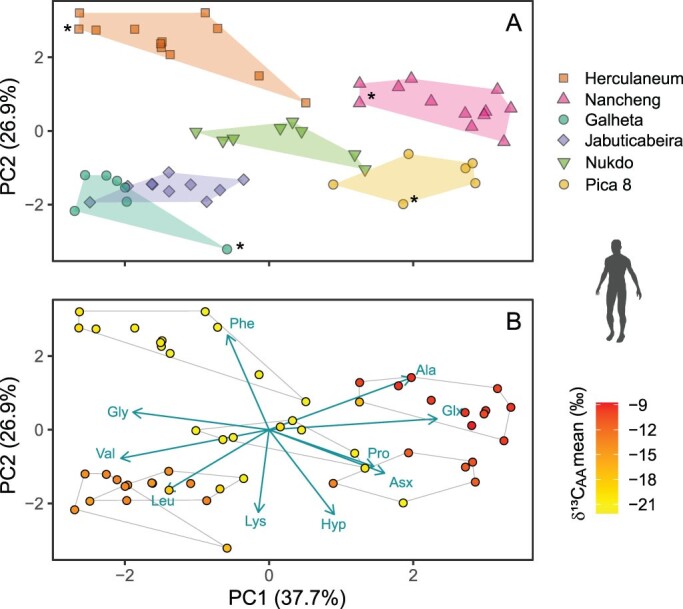
Principal component analysis of compiled δ^13^C_amino acid_ data from human archeological bone or tendon collagen. (a) The principal component scores based on mean-centered δ^13^C values of four essential amino acids (leucine, lysine, phenylalanine, valine) and six nonessential amino acids (alanine, aspartatic acid, glutamic acid, glycine, hydroxyproline, proline). (b) A mirror of panel (a) but color coded according to their mean δ^13^C_amino acid_ values. The median discriminant values of six sites based on the first three principal component (PC) scores (accounting for 75.5% of the variation) were significantly different (Pillai's trace = 2.14, F(***15,157***) = 24.4, p < .001). The convex hulls represent the maximum range of the PC1 and PC2 scores of each group, and the arrows represent the relative weightings of the independent variables for creating the principal component analysis. The symbols marked with asterisks denote outlier individuals with deviating linear discriminant scores (see figure 7) or δ^13^C_amino acid_ baselines. References: Jabuticabeira II, Piacaguera, and Galheta IV (Brazil, 6700 to 1000 calibrated years before the present; Colonese et al. 2014), Herculaneum (Rome, 79 CE; Soncin et al. 2021), Nancheng (China, 2000–1600 BCE; Ma et al. 2021), Nukdo shell midden (Korea, 550 BCE to 1 CE; Choy et al. 2010), and Pica 8 (Peru, 1050–500 calibrated years before the present; Mora et al. 2018). See supplemental appendix S2 for sample information. Image: Silhouettes from PhyloPic (http://phylopic.org) under a Creative Commons license.

To further explore how data from human osteological remains can contribute to an increased interpretive power of different *δ*^13^C_amino acid_ based dietary estimates, we compiled published *δ*^13^C_amino acid_ data of humans from archeological sites applying the criteria that the data contained at least four essential amino acid variables (leucine, lysine, phenylalanine, and valine). In addition to the Herculaneum and Nancheng individuals, we compiled data from four other sites: The first two sites are Jabuticabeira II, Piacaguera, and Galheta IV (6700 to 1000 calibrated years before the present), in Brazil (Colonese et al. 2014), where humans mainly subsisted on a mixture of aquatic resources and plant foods. The other two sites are the Nukdo shell midden (Korea, 550 BCE to 1 CE; Choy et al. 2010), where humans subsisted on mixed marine and C_3_ terrestrial diets, and Pica 8 (Peru, 1050–500 ago), where humans mostly subsisted on a maize-dominated diet (Mora et al. 2018). Based on the PCA of the four available essential amino acids and six nonessential amino acids, we found that the six populations clustered separately but with the two Brazilian populations grouping adjacently (figure 8a). Both PC1 and PC2 are needed to separate consumers with marine- and terrestrial-dominated diets. The fact that several of the Nukdo individuals fall next to the two Brazilian groups is likely indicative of a high marine protein intake. The disparity in principal components scores and *δ*^13^C_amino acid_ baselines between the Brazilian and the Herculaneum individuals (figure 8b) supports the fact that marine fish made a smaller dietary contribution in the latter case (Colonese et al. 2014, Soncin et al. 2021). In terms of phenylalanine versus valine separating terrestrial from aquatic consumers (Honch et al. 2012, Webb et al. 2017), our data suggest phenylalanine versus the three using essential amino acids (valine, leucine, and lysine) is a more powerful approach for separating these dietary groups.

The comparative analysis groups the Herculaneum outlier with other individuals from the same site suggesting that analytical uncertainty of an essential amino acid variable explained its outlier position in the LDA. This finding reinforces the importance of analyzing as many amino acid variables as possible when making robust dietary inferences. The comparative analysis also bins the Nancheng outlier with other individuals from the same site, but it remains more ^13^C depleted than the others. The most parsimonious explanation for this and the two ^13^C-depleted Galheta and Pica 8 individuals (marked with asterisks in figure 8a) is a contribution of C_3_-derived proteins. In terms of lysine and phenylalanine separating the two C_4_-dominated biomes, Nancheng and Pica 8, it may be relevant to investigate how protein quality and food preparation affect lysine to phenylalanine *δ*^13^C spacing. Lysine is the first limiting essential amino acid and phenylalanine the last limiting essential amino acid in both maize and pearl millet (Anitha et al. 2020, Wiedemair et al. 2020). For this reason, human *δ*^13^C_lysine_ values are likely to be much more sensitive than *δ*^13^C_phenylalanine_ values to protein supplementation from animal foods and food processing increasing digestibility. With regards to the nonessential amino acids, it is noteworthy that the eigenvectors of glycine and alanine point in opposite directions, confirming observations from human epidemiological studies and animal feeding trials that dietary macronutrients are routed differently to these two glycolytic amino acids (Wang et al. 2019, Yun et al. 2020). These and potentially many other observations pertaining to the relationship among amino acid variables highlight how comparative studies based on archeological humans with relatively robust dietary contexts can provide clues on how to interpret *δ*^13^C_amino acid_ data.

## Perspectives for hominin studies

Bulk *δ*^13^C and *δ*^15^N analysis of ecological and archeological proteinaceous tissues has long served as an important avenue for exploring the diets and life histories of hominins (Sehrawat and Kaur 2017). However, issues of equifinality, influences of environmental variation, and a general coarseness of interpretation have demanded new biomarkers for discerning variation in resource use, the consumption of different macronutrients, and interorganism relationships on local, regional, and even global scales. We hope to have demonstrated that *δ*^13^C_amino acid_ and *δ*^15^N_amino acid_ approaches have such a potential. Although applications remain limited in archeology, anthropology, and (paleo)ecology, the joint application of these compound-specific isotopic methodologies offers the opportunity of pinpointing trends of dietary reliance, perhaps even to individual resources in some cases, and determining metabolic patterns and nutritional deficiencies. For these methods to reach their full potential and to be used in a proper and uniform manner, it is essential that a wider, global multidisciplinary audience is aware of both their promise and current limitations.

As with many novel methodological tracer approaches, the enthusiastic application of *δ*^13^C_amino acid_ and *δ*^15^N_amino acid_ approaches should be tempered by a need for consistency in preparation, measurement, and correction for added carbon during derivatization and for the use of reference standards and samples (Roberts et al. 2018, Meier-Augenstein and Schimmelmann 2019). Interlaboratory comparison and shared protocols are essential, because the potential for *δ*^13^C_amino acid_ and *δ*^15^N_amino acid_ variation as a result of derivatization and measurement methodologies are becoming increasingly apparent (Ohkouchi et al. 2017, Yarnes and Herszage 2017), and compound-specific practitioners should follow the work done on bulk isotopic analysis of proteinaceous materials in this regard (Brand et al. 2014). To facilitate interlaboratory comparisons of amino acid stable isotope ratio data, it would be particularly advantageous if, with every batch of archeological samples, the research community agreed to include a couple of globally available collagen samples extracted from modern faunal bones. Ideally, these samples should encompass the naturally occurring range of amino acid stable isotope ratio values (e.g., a C_3_ herbivore and a marine carnivore, respectively). More open, thorough discussions on the topic of analytical issues are essential if growing *δ*^13^C_amino acid_ and *δ*^15^N_amino acid_ data sets in archeology are going to be comparable and useful for future meta-analyses and building understandings of how modern variation can be related to archeological problems.

Our multiple variate analysis of amino acid stable isotope ratio data demonstrates the importance of embracing as many amino acid variables as possible for robust dietary inferences and the need for expanding training and comparative data. It is crucial that researchers publish and perform quality control on as many amino acid variables as possible. In this way, larger reference data sets and multivariate evaluations of patterns can help to explore the full utility of *δ*^13^C_amino acid_ and *δ*^15^N_amino acid_ variability. With regards to *δ*^13^C, the nonessential amino acids are more complex to interpret than the essential amino acids, but they do open the door for the development of a series of additional biomarker approaches to diet reconstruction, such as inferring macromolecular composition (Larsen et al. 2022). In the case of *δ*^15^N, source-trophic pairs, such as proline and lysine, or glutamic acid and lycine, may provide more consistent trophic position estimates for hominins than the commonly used glutamic acid–phenylalanine pair. Moving beyond trophic position could potentially yield a more holistic understanding of the nutritional status of consumers, such as employing *δ*^15^N_threonine_ as a biomarker for protein intake (Fuller and Petzke 2017). The aspiration to apply amino acid stable isotope ratio analyses for paleodietary reconstruction should be juxtaposed against their relatively high analytical costs. However, ongoing advances in analytical approaches, an expansion of laboratories with the capacity to measure *δ*^13^C_amino acid_ and *δ*^15^N_amino acid_, and emerging exposure in archeological and anthropological disciplines promise to see amino acid stable isotope ratios become an increasingly regular part of paleodietary and paleoecological research agendas.

Data from modern populations can increase our ability to associate particular amino acid stable isotope ratio patterns with biomes and regions, but dietary homogenization and the increasing globalization of the food supply is making such efforts increasingly difficult. For instance, the contrasts between modern and ancient gut microbiomes (Wibowo et al. 2021) remain uncertain with regards to amino acid stable isotope ratio patterns. Despite these caveats, modern amino acid stable isotope ratio studies will be crucial for developing taxon or biome specific fingerprints that can be used as baselines for the interpretation of human data in the past. Observational studies of humans and other primates will also be important for exploring how amino acid stable isotope ratio variability is related to physiological and dietary factors. Besides applying *δ*^13^C_nonessential amino acid_ values as biomarkers of particular foodstuffs (Choy et al. 2013, Yun et al. 2020, Johnson et al. 2021), researchers are also investigating the extent *δ*^13^C_amino acid_ values can inform about biometric traits, such as body mass index, age, and sex (Jackson et al. 2015, Matos and Jackson 2020). More feeding experiments on pigs and other mammalian model species are needed to improve contextualization of omnivore-specific *δ*^13^C_amino acid_ patterns (Edgar Hare et al. 1991, Webb et al. 2017). This is particularly true for nutritional stressors, such as starvation and a lack or an excess of dietary proteins (Doi et al. 2017, Fry and Carter 2019). Moreover, a growing number of projects using modern human and primate hair and teeth, within appropriate ethically designed studies, have been used to better understand patterns of bulk isotope variation connected to the environment (Macho and Lee-Thorp 2014), season (Oelze 2016), weaning (Dailey-Chwalibóg et al. 2020), and physiological stress (D'Ortenzio et al. 2015, Crowley et al. 2016), providing useful models for future amino acid stable isotope ratio research. Much can be learned as well from human studies applying bulk stable isotope ratios as health indicators (Petzke et al. 2010, O'Brien 2015).

The adage that something is greater than the sum of its parts is particularly true for archeological amino acid stable isotope ratio data. The literacy of amino acid stable isotope ratio data can be enhanced by tying them to contextual information such as the environment humans lived in, social conditions and the artefacts they left behind, and then comparing this information across different populations. For example, as our compilation data set shows, determining the amino acid stable isotope ratios of humans living in different regions with different observed diets (based on archeobotany, archeozoology, and, in some cases, historical records) can provide a point of exploration as to how spacing of *δ*^13^C and *δ*^15^N among different amino acids relates to diet or ecology. This may, in turn, provide targeted areas for future research. Similarly, a combination of *δ*^13^C_amino acid_ and *δ*^15^N_amino acid_ from archeological contexts with DNA studies of the microbiome and other multidisciplinary approaches to food preparation may provide insights into how specific biological digestive processes and cultural manipulation of foodstuffs (e.g., fermentation) might influence *δ*^13^C_amino acid_ and *δ*^15^N_amino acid_ variability. As we demonstrated in the present article, amino acid stable isotope ratio analyses of hominin remains, where preservation conditions allow, hold a vast potential for advancing and nuancing our understanding of past human–environment interactions. A deep understanding of our past is important, because increasing social complexity and implementation of new technologies in human societies have not only shaped the diet of own species but have also altered the habitats and resource base of other species (Moll et al. 2021). To fulfill the promise of amino acid stable isotope ratios, both modern and archeological data sets will be essential for providing important reference points for multivariate modeling and probing of isotopic and dietary variation.

## Supplementary Material

biac028_Supplemental_Files

## Author Biographical

Thomas Larsen (larsen@shh.mpg.de), Ricardo Fernandes, Yiming V. Wang, and Patrick Roberts are affiliated with the Department of Archaeology at the Max Planck Institute for the Science of Human History, in Jena, Germany. Ricardo Fernandes is affiliated with the School of Archaeology at the University of Oxford, in Oxford, England, in the United Kingdom, and with the Faculty of Arts at Masaryk University, in the Czech Republic. Patrick Roberts is affiliated with the School of Social Sciences at the University of Queensland, in St Lucia, Queensland, Australia.
