# Supplementary material for: Reconstructing Hominin Diets with Stable Isotope Analysis of Amino Acids: New Perspectives and Future Directions
Source: Bioscience. 2022 May 23;72(7):618–37. doi: 10.1093/biosci/biac028 (PMC9236875; doi:10.1093/biosci/biac028)
Supplement: biac028_Supplemental_Files [file biac028_supplemental_files.zip › AppendixA.docx]

**Appendix A**

**Larsen et al.: Reconstructing hominin diets with stable isotope analysis of amino acids – new perspectives and future directions**

**Statistics**

Differences in isotope values among groups were tested with one-way ANOVA with Tukey’s HSD test (R: *aov; TukeyHSD*, α=0.05). To compare δ^13^C_AA_ patterns among faunal and human groups and classify diet sources, we applied principal component analysis (PCA) (R: *prcomp*) and linear discriminant function analysis (LDA) (R: *MASS*). We used covariance matrix PCA that preserves variance as the range and scale of variables are in the same units of measure. Based on the first and second PC/ LD scores, we used two different approaches to visualize predefined groups; 95% prediction ellipses visualize variability relative to the group centroid, and convex hulls visualize the amount of space taken up by a given group. We applied Multivariate Analysis of Variance (MANOVA, R: *manova*) in conjunction with Pillai’s trace to test the null hypothesis that groups have a common centroid in a dependent variable vector space. A rejection of this hypothesis entails that the groups have significantly different δ^13^C_EAA_ patterns or fingerprints. All data for multivariate comparisons were first assessed for homogeneity of variance by using Fligner-Killeen tests (R: *fligner.test*) and visually checked for departures from normality on Q-Q plots. R version 3.6.3 was used for statistical analyses (R-Development-Core-Team 2020) and ggplot2 for figure production (Wickham 2016).

**References**

R-Development-Core-Team. 2020. R: A language and environment for statistical computing. Vienna, Austria: R Foundation for Statistical Computing.

Wickham H. 2016. ggplot2: Elegant Graphics for Data Analysis. Springer International Publishing.
