# Supplementary material for: Reconstructing Hominin Diets with Stable Isotope Analysis of Amino Acids: New Perspectives and Future Directions
Source: Bioscience. 2022 May 23;72(7):618–37. doi: 10.1093/biosci/biac028 (PMC9236875; doi:10.1093/biosci/biac028)
Supplement: biac028_Supplemental_Files [file biac028_supplemental_files.zip › AppendixB.pdf]

Data availability	Identifier	Taxa	Tissue	Site	Date	Diet	Figure
Campbell et al. 2017	G-27641	*Glossophaga sorici handleyi*	Hair	Guatemala	8-May-83	Carnivorous	2
Campbell et al. 2017	G-27637	*Glossophaga sorici handleyi*	Hair	Guatemala	8-May-83	Carnivorous	2
Campbell et al. 2017	G-27638	*Glossophaga sorici handleyi*	Hair	Guatemala	8-May-83	Carnivorous	2
Campbell et al. 2017	G-27645	*Myotis vivesi*	Hair	Mexico	1-May-48	Piscivorous	2
Campbell et al. 2017	G-27642	*Myotis vivesi*	Hair	Mexico	1-May-48	Carnivorous	2
Campbell et al. 2017	G-27644	*Myotis vivesi*	Hair	Mexico	1-May-48	Piscivorous	2
Campbell et al. 2017	G-27643	*Myotis vivesi*	Hair	Mexico	1-May-48	Piscivorous	2
Campbell et al. 2017	G-27648	*Vampyrum spectrum*	Hair	Panama	21-Jan-63	Sanguivorous	2
Campbell et al. 2017	G-27665	*Diphylla ecaudata*	Hair	Panama	8-Feb-64	Sanguivorous	2
Campbell et al. 2017	G-27655	*Desmodus rotundus*	Hair	Panama	23-Jan-60	Frugivorous	2
Campbell et al. 2017	G-27649	*Vampyrum spectrum*	Hair	Panama	23-Jan-63	Sanguivorous	2
Campbell et al. 2017	G-27650	*Vampyrum spectrum*	Hair	Panama	12-Mar-63	Sanguivorous	2
Campbell et al. 2017	G-27647	*Vampyrum spectrum*	Hair	Panama	20-Jan-63	Piscivorous	2
Campbell et al. 2017	G-27664	*Diphylla ecaudata*	Hair	Panama	8-Feb-64	Sanguivorous	2
Campbell et al. 2017	G-27667	*Diphylla ecaudata*	Hair	Panama	16-Feb-64	Sanguivorous	2
Campbell et al. 2017	G-27653	*Desmodus rotundus*	Hair	Panama	23-Jan-60	Frugivorous	2
Campbell et al. 2017	G-27654	*Desmodus rotundus*	Hair	Panama	23-Jan-60	Frugivorous	2
Campbell et al. 2017	G-27441	*Lasionycteris noctivagans*	Hair	United States	9-Sep-09	Insectivorous	2
Campbell et al. 2017	G-27452	*Lasionycteris noctivagans*	Hair	United States	19-Sep-09	Insectivorous	2
Campbell et al. 2017	G-27450	*Lasiurus cinereus*	Hair	United States	3-Sep-10	Insectivorous	2
Campbell et al. 2017	G-27472	*Lasionycteris noctivagans*	Hair	United States	9-May-09	Insectivorous	2
Campbell et al. 2017	G-27454	*Lasiurus cinereus*	Hair	United States	25-Jul-11	Insectivorous	2
Campbell et al. 2017	G-27455	*Lasiurus cinereus*	Hair	United States	n/a	Insectivorous	2
Campbell et al. 2017	G-27633	*Trachops cirrhosus cirrhosus*	Hair	Venezuela	3-Apr-67	Carnivorous	2
Campbell et al. 2017	G-27635	*Trachops cirrhosus cirrhosus*	Hair	Venezuela	3-Apr-67	Carnivorous	3
Campbell et al. 2017	G-27659	*Artibeus jamaicensis*	Hair	Venezuela	19-Aug-65	Frugivorous	3
Campbell et al. 2017	G-27646	*Trachops cirrhosus cirrhosus*	Hair	Venezuela	3-Apr-67	Piscivorous	3
Campbell et al. 2017	G-27658	*Artibeus jamaicensis*	Hair	Venezuela	19-Aug-65	Frugivorous	3
Campbell et al. 2017	G-27661	*Artibeus jamaicensis*	Hair	Venezuela	20-Aug-65	Frugivorous	3
Schwartz-Narbonne et al. 2015	YT129	*Equus caballus*	Bone	Old Crow, Yukon	Pleistocene	C3 herbivore	3
Schwartz-Narbonne et al. 2015	YT130	*Equus caballus*	Bone	Old Crow, Yukon	Pleistocene	C3 herbivore	3
Schwartz-Narbonne et al. 2015	YT131	*Equus caballus*	Bone	Old Crow, Yukon	Pleistocene	C3 herbivore	3
Schwartz-Narbonne et al. 2015	YT132	*Equus caballus*	Bone	Old Crow, Yukon	Pleistocene	C3 herbivore	3
Schwartz-Narbonne et al. 2015	YT133	*Equus caballus*	Bone	Old Crow, Yukon	Pleistocene	C3 herbivore	3
Naito et al. 2013	PAM1700	*Equus caballus*	Bone	Pont d'Ambon, France	Epipalaeolithic (13000-9500 BP)	C3 herbivore	3
Naito et al. 2013	PAM700	*Equus caballus*	Bone	Pont d'Ambon, France	Epipalaeolithic (13000-9500 BP)	C3 herbivore	3
Naito et al. 2013	SC3900	*Equus caballus*	Bone	Noyen-sur-Seine, Belgium	Mesolithic (8000 BP)	C3 herbivore	3
Naito et al. 2013	SC4200	*Equus caballus*	Bone	Noyen-sur-Seine, Belgium	Mesolithic (8000 BP)	C3 herbivore	3
Naito et al. 2013	SC4300	*Equus caballus*	Bone	Noyen-sur-Seine, Belgium	Mesolithic (8000 BP)	C3 herbivore	3

Data availability	Identifier	Taxa	Tissue	Site	Date	Diet	Figure
Jarman et al. 2017	RN026	*Homo sapiens*	Phalanx	Ahu Tepeu E-13, grave 2	c. 1400 AD	C3/marine omnivore	4
Jarman et al. 2017	RN033	*Homo sapiens*	Phalanx	Ahu Nau Nau/Trench K	c. 1400 AD	C3/marine omnivore	4
Jarman et al. 2017	RN035	*Homo sapiens*	Rib fragme	Ahu Nau Nau/Trench K	c. 1400 AD	C3/marine omnivore	4
Jarman et al. 2017	RN036	*Homo sapiens*	Rib fragme	Ahu Nau Nau/Trench K	c. 1400 AD	C3/marine omnivore	4
Jarman et al. 2017	RN037	*Homo sapiens*	Rib fragme	Ahu Nau Nau/Trench K	c. 1400 AD	C3/marine omnivore	4
Jarman et al. 2017	RN039	*Homo sapiens*	Rib fragme	Ahu Nau Nau/Trench K	c. 1400 AD	C3/marine omnivore	4
Jarman et al. 2017	RN041	*Homo sapiens*	Rib fragme	Ahu Nau Nau/Trench K	c. 1400 AD	C3/marine omnivore	4
Choy et al. 2010	SEVA-3931	*Zalophus japonicus*	Bone	Nukdo shell midden, Korea	Late Bronze Age (BC 550–1 AD)	Marine carnivore	5
Choy et al. 2010	SEVA-1720	*Thunnus*	Bone	Nukdo shell midden, Korea	Neolithic (BC 3500–2500)	Marine carnivore	5
Choy et al. 2010	SEVA-1721	*Zalophus japonicus*	Bone	Nukdo shell midden, Korea	Neolithic (BC 3500–2500)	Marine carnivore	5
Choy et al. 2010	SEVA-1732	*Cervus nippon*	Bone	Nukdo shell midden, Korea	Neolithic (BC 3500–2500)	C3 herbivore	5
Choy et al. 2010	SEVA-1735	*Sus scrofa*	Bone	Nukdo shell midden, Korea	Neolithic (BC 3500–2500)	C3 herbivore	5
Choy et al. 2010	SEVA-3918	*Delphinidae*	Bone	Nukdo shell midden, Korea	Neolithic (BC 3500–2500)	Marine carnivore	5
Choy et al. 2010	SEVA-3920	*Zalophus japonicus*	Bone	Nukdo shell midden, Korea	Neolithic (BC 3500–2500)	Marine carnivore	5
Ma et al. 2021	H143:4	Cervidae	Bone	Nancheng, China	2000-1600 BC	C3 herbivore	5
Ma et al. 2021	H62:2	Cervidae	Bone	Nancheng, China	2000-1600 BC	C3 herbivore	5
Ma et al. 2021	H122:3	Bos	Bone	Nancheng, China	2000-1600 BC	C4 herbivore	5
Ma et al. 2021	H23:2	Bos	Bone	Nancheng, China	2000-1600 BC	C4 herbivore	5
Ma et al. 2021	H2:1	Ovis	Bone	Nancheng, China	2000-1600 BC	C4 herbivore	5
Ma et al. 2021	H122:1	Ovis	Bone	Nancheng, China	2000-1600 BC	C4 herbivore	5
Ma et al. 2021	H52:1	Capra	Bone	Nancheng, China	2000-1600 BC	C4 herbivore	5
Ma et al. 2021	H28: 3	Capra	Bone	Nancheng, China	2000-1600 BC	C4 herbivore	5
Ma et al. 2021	H52:2	Sus	Bone	Nancheng, China	2000-1600 BC	C4 herbivore	5
Soncin et al. 2021	EF7OC	Ovis	Bone	Herculaneum, Italy	79 AD	C3 herbivore	5
Soncin et al. 2021	F8SG	Ovis	Bone	Herculaneum, Italy	79 AD	C3 herbivore	5
Soncin et al. 2021	EF8BOS	Bos	Bone	Herculaneum, Italy	79 AD	C3 herbivore	5
Soncin et al. 2021	EF10OC	Ovis	Bone	Herculaneum, Italy	79 AD	C3 herbivore	5
Soncin et al. 2021	PSC1	Bos	Bone	Porta Stabia - Pompeii, Italy	Early I BC	C3 herbivore	5
Soncin et al. 2021	PSC2	Bos	Bone	Porta Stabia - Pompeii, Italy	Early I BC	C3 herbivore	5
Soncin et al. 2021	PSG2	Capreolus	Bone	Porta Stabia - Pompeii, Italy	Early I AD	C3 herbivore	5
Soncin et al. 2021	PSP3	Sus	Bone	Porta Stabia - Pompeii, Italy	Early I AD	C3 herbivore	5
Soncin et al. 2021	PSP4	Sus	Bone	Porta Stabia - Pompeii, Italy	Early I AD	C3 herbivore	5
Soncin et al. 2021	PSP5	Sus	Bone	Porta Stabia - Pompeii, Italy	Early I AD	C3 herbivore	5
Soncin et al. 2021	HSSPI	Sparidae	Bone	Porta Stabia - Pompeii, Italy	Early I AD	C3 herbivore	5
Soncin et al. 2021	HSSPII	Sparidae	Bone	Porta Stabia - Pompeii, Italy	Early I AD	C3 herbivore	5
Soncin et al. 2021	PSSC2	Scombridae	Bone	Porta Stabia - Pompeii, Italy	Early I AD	C3 herbivore	5
Soncin et al. 2021	VESH1	Capra	Bone	Velia, Italy	I - II AD	C3 herbivore	5
Soncin et al. 2021	VEDE1	Capreolus	Bone	Velia, Italy	I - II AD	C3 herbivore	5
Soncin et al. 2021	PSSPI	Sparidae	Bone	House of the Surgeon - Pompeii, Italy	III BC - I AD	Marine carnivore	5

Data availability	Identifier	Taxa	Tissue	Site	Date	Diet	Figure
Soncin et al. 2021	HSSSQ	*Squatina squatina*	Bone	House of the Surgeon - Pompeii, Italy	Early I AD	Marine carnivore	5
Soncin et al. 2021	HSSSA	*Sarda sarda*	Bone	House of the Surgeon - Pompeii	III BC - I AD	Marine carnivore	5
Soncin et al. 2021	HSLA	Labridae	Bone	House of the Surgeon - Pompeii	III BC - I AD	Marine carnivore	5
Soncin et al. 2021	ABF3	*Argyrosomus regius*	Bone	Albarracín, Spain	X-XII AD	Marine carnivore	5
Ma et al. 2021	M12	*Homo sapiens*	Bone	Nancheng, China	2000-1600 BC	C4 omnivore	5,6
Ma et al. 2021	M13	*Homo sapiens*	Bone	Nancheng, China	2000-1600 BC	C4 omnivore	5,6
Ma et al. 2021	M14	*Homo sapiens*	Bone	Nancheng, China	2000-1600 BC	C4 omnivore	5,6
Ma et al. 2021	M28	*Homo sapiens*	Bone	Nancheng, China	2000-1600 BC	C4 omnivore	5,6
Ma et al. 2021	M43	*Homo sapiens*	Bone	Nancheng, China	2000-1600 BC	C4 omnivore	5,6
Ma et al. 2021	M55	*Homo sapiens*	Bone	Nancheng, China	2000-1600 BC	C4 omnivore	5,6
Ma et al. 2021	M56	*Homo sapiens*	Bone	Nancheng, China	2000-1600 BC	C4 omnivore	5,6
Ma et al. 2021	M57	*Homo sapiens*	Bone	Nancheng, China	2000-1600 BC	C4 omnivore	5,6
Ma et al. 2021	M70	*Homo sapiens*	Bone	Nancheng, China	2000-1600 BC	C4 omnivore	5,6
Ma et al. 2021	M71	*Homo sapiens*	Bone	Nancheng, China	2000-1600 BC	C4 omnivore	5,6
Ma et al. 2021	M75	*Homo sapiens*	Bone	Nancheng, China	2000-1600 BC	C4 omnivore	5,6
Ma et al. 2021	M77	*Homo sapiens*	Bone	Nancheng, China	2000-1600 BC	C4 omnivore	5,6
Soncin et al. 2021	F10i11	*Homo sapiens*	Bone	Herculaneum (Fornici 10), Italy	79 AD	C3/marine omnivore	5,6
Soncin et al. 2021	F10i16	*Homo sapiens*	Bone	Herculaneum (Fornici 10), Italy	79 AD	C3/marine omnivore	5,6
Soncin et al. 2021	F10i28	*Homo sapiens*	Bone	Herculaneum (Fornici 10), Italy	79 AD	C3/marine omnivore	5,6
Soncin et al. 2021	F12I3	*Homo sapiens*	Bone	Herculaneum (Fornici 12), Italy	79 AD	C3/marine omnivore	5,6
Soncin et al. 2021	F7I7	*Homo sapiens*	Bone	Herculaneum (Fornici 7), Italy	79 AD	C3/marine omnivore	5,6
Soncin et al. 2021	F8I7	*Homo sapiens*	Bone	Herculaneum (Fornici 8), Italy	79 AD	C3/marine omnivore	5,6
Soncin et al. 2021	F9i9	*Homo sapiens*	Bone	Herculaneum (Fornici 9), Italy	79 AD	C3/marine omnivore	5,6
Soncin et al. 2021	F9i13	*Homo sapiens*	Bone	Herculaneum (Fornici 9), Italy	79 AD	C3/marine omnivore	5,6
Soncin et al. 2021	F10I14	*Homo sapiens*	Bone	Herculaneum (Fornici 10), Italy	79 AD	C3/marine omnivore	5,6
Soncin et al. 2021	F10I20	*Homo sapiens*	Bone	Herculaneum (Fornici 10), Italy	79 AD	C3/marine omnivore	5,6
Soncin et al. 2021	F10I22	*Homo sapiens*	Bone	Herculaneum (Fornici 10), Italy	79 AD	C3/marine omnivore	5,6
Soncin et al. 2021	F12I23	*Homo sapiens*	Bone	Herculaneum (Fornici 12), Italy	79 AD	C3/marine omnivore	5,6
Choy et al. 2010	SEVA-1939	*Homo sapiens*	Bone	Nukdo shell midden, Korea	Late Bronze Age (BC 550–1 AD)	C3/marine omnivore	6
Choy et al. 2010	SEVA-1943	*Homo sapiens*	Bone	Nukdo shell midden, Korea	Late Bronze Age (BC 550–1 AD)	C3/marine omnivore	6
Choy et al. 2010	SEVA-1951	*Homo sapiens*	Bone	Nukdo shell midden, Korea	Late Bronze Age (BC 550–1 AD)	C3/marine omnivore	6
Choy et al. 2010	SEVA-1954	*Homo sapiens*	Bone	Nukdo shell midden, Korea	Late Bronze Age (BC 550–1 AD)	C3/marine omnivore	6
Choy et al. 2010	SEVA-1964	*Homo sapiens*	Bone	Nukdo shell midden, Korea	Late Bronze Age (BC 550–1 AD)	C3/marine omnivore	6
Choy et al. 2010	SEVA-1968	*Homo sapiens*	Bone	Nukdo shell midden, Korea	Late Bronze Age (BC 550–1 AD)	C3/marine omnivore	6
Choy et al. 2010	SEVA-1971	*Homo sapiens*	Bone	Nukdo shell midden, Korea	Late Bronze Age (BC 550–1 AD)	C3/marine omnivore	6
Choy et al. 2010	SEVA-1976	*Homo sapiens*	Bone	Nukdo shell midden, Korea	Late Bronze Age (BC 550–1 AD)	C3/marine omnivore	6
Colonese et al. 2014	Jab_II_1B	*Homo sapiens*	Bone	Jabuticabeira II, Piacaguera, Brazil	6700-1700 cal BP	C3/marine omnivore	6
Colonese et al. 2014	Jab_II_8	*Homo sapiens*	Bone	Jabuticabeira II, Piacaguera, Brazil	6700-1700 cal BP	C3/marine omnivore	6
Colonese et al. 2014	Jab_II_35A	*Homo sapiens*	Bone	Jabuticabeira II, Piacaguera, Brazil	6700-1700 cal BP	C3/marine omnivore	6

Data availability	Identifier	Taxa	Tissue	Site	Date	Diet	Figure
Colonese et al. 2014	Jab_II_107	*Homo sapiens*	Bone	Jabuticabeira II, Piacaguera, Brazil	6700-1700 cal BP	C3/marine omnivore	6
Colonese et al. 2014	Jab_II_108	*Homo sapiens*	Bone	Jabuticabeira II, Piacaguera, Brazil	6700-1700 cal BP	C3/marine omnivore	6
Colonese et al. 2014	Jab_II_115B	*Homo sapiens*	Bone	Jabuticabeira II, Piacaguera, Brazil	6700-1700 cal BP	C3/marine omnivore	6
Colonese et al. 2014	Jab_II_118	*Homo sapiens*	Bone	Jabuticabeira II, Piacaguera, Brazil	6700-1700 cal BP	C3/marine omnivore	6
Colonese et al. 2014	Jab_II_17C	*Homo sapiens*	Bone	Jabuticabeira II, Piacaguera, Brazil	6700-1700 cal BP	C3/marine omnivore	6
Colonese et al. 2014	Jab_II_24A	*Homo sapiens*	Bone	Jabuticabeira II, Piacaguera, Brazil	6700-1700 cal BP	C3/marine omnivore	6
Colonese et al. 2014	Jab_II_102	*Homo sapiens*	Bone	Jabuticabeira II, Piacaguera, Brazil	6700-1700 cal BP	C3/marine omnivore	6
Colonese et al. 2014	G_IV_1	*Homo sapiens*	Bone	Galheta IV, Brazil	6700-1700 cal BP	C3/marine omnivore	6
Colonese et al. 2014	G_IV_2	*Homo sapiens*	Bone	Galheta IV, Brazil	6700-1700 cal BP	C3/marine omnivore	6
Colonese et al. 2014	G_IV_4	*Homo sapiens*	Bone	Galheta IV, Brazil	6700-1700 cal BP	C3/marine omnivore	6
Colonese et al. 2014	G_IV_5	*Homo sapiens*	Bone	Galheta IV, Brazil	6700-1700 cal BP	C3/marine omnivore	6
Colonese et al. 2014	G_IV_6	*Homo sapiens*	Bone	Galheta IV, Brazil	6700-1700 cal BP	C3/marine omnivore	6
Colonese et al. 2014	G_IV_9	*Homo sapiens*	Bone	Galheta IV, Brazil	6700-1700 cal BP	C3/marine omnivore	6
Colonese et al. 2014	G_IV_7	*Homo sapiens*	Bone	Galheta IV, Brazil	6700-1700 cal BP	C3/marine omnivore	6
Mora et al. 2018	SI-T74	*Homo sapiens*	Tendon	Pica 8, Tarapacá, Chile	1050-500 BP	C4 omnivore	6
Mora et al. 2018	SD-T24	*Homo sapiens*	Tendon	Pica 8, Tarapacá, Chile	1050-500 BP	C4 omnivore	6
Mora et al. 2018	SI-T32	*Homo sapiens*	Tendon	Pica 8, Tarapacá, Chile	1050-500 BP	C4 omnivore	6
Mora et al. 2018	SE-T3	*Homo sapiens*	Tendon	Pica 8, Tarapacá, Chile	1050-500 BP	C4 omnivore	6
Mora et al. 2018	SF-T4	*Homo sapiens*	Tendon	Pica 8, Tarapacá, Chile	1050-500 BP	C4 omnivore	6
Mora et al. 2018	SI-T3	*Homo sapiens*	Tendon	Pica 8, Tarapacá, Chile	1050-500 BP	C4 omnivore	6

References

Campbell, C.J., Nelson, D.M., Ogawa, N.O., Chikaraishi, Y. & Ohkouchi, N. (2017) Trophic position and dietary breadth of bats revealed by nitrogen isotopic composition of amino acids. *Scientific Reports,* **7,** 15932.

Choy, K., Smith, C.I., Fuller, B.T. & Richards, M.P. (2010) Investigation of amino acid $\delta^{13}C$ signatures in bone collagen to reconstruct human palaeodiets using liquid chromatography-isotope ratio mass spectrometry. *Geochimica et Cosmochimica Acta,* **74,** 6093-6111.

Colonese, A.C., Collins, M., Lucquin, A., Eustace, M., Hancock, Y., de Almeida Rocha Ponzoni, R., Mora, A., Smith, C., DeBlasis, P., Figuti, L., Wesolowski, V., Plens, C.R., Eggers, S., de Farias, D.S.E., Gledhill, A. & Craig, O.E. (2014) Long-Term Resilience of Late Holocene Coastal Subsistence System in Southeastern South America. *Plos One,* **9,** e93854.

Jarman, C.L., Larsen, T., Hunt, T., Lipo, C., Solsvik, R., Wallsgrove, N., Ka'apu-Lyons, C., Close, H.G. & Popp, B.N. (2017) Diet of the prehistoric population of Rapa Nui (Easter Island, Chile) shows environmental adaptation and resilience. American Journal of Physical Anthropology, 164, 343–361.

Ma, Y., Grimes, V., Van Biesen, G., Shi, L., Chen, K., Mannino, M.A. & Fuller, B.T. (2021) Aminoisoscapes and palaeodiet reconstruction: New perspectives on millet-based diets in China using amino acid δ13C values. *Journal of Archaeological Science,* **125,** 105289.

Mora, A., Pacheco, A., Roberts, C. & Smith, C. (2018) Pica 8: Refining dietary reconstruction through amino acid $\delta^{13}C$ analysis of tendon collagen and hair keratin. *Journal of Archaeological Science,* **93,** 94-109.

Naito, Y.I., Bocherens, H., Chikaraishi, Y., Drucker, D.G., Wißing, C., Yoneda, M. & Ohkouchi, N. (2016a) An overview of methods used for the detection of aquatic resource consumption by humans: Compound-specific $d^{15}N$ analysis of amino acids in archaeological materials. *Journal of Archaeological Science: Reports,* **6,** 720-732.

Schwartz-Narbonne, R., Longstaffe, F.J., Metcalfe, J.Z. & Zazula, G. (2015) Solving the woolly mammoth conundrum: amino acid ^{15}N-enrichment suggests a distinct forage or habitat. *Scientific Reports,* **5,** 9791.

Soncin, S., Talbot, H.M., Fernandes, R., Harris, A., Tersch, M.v., Robson, H.K., Bakker, J.K., Alexander, K.K.R.M., Ellis, S., Thompson, G., Amoretti, V., Osanna, M., Caso, M., Sirano, F., Fattore, L., Colonese, A.C., Garnsey, P., Bondioli, L. & Craig, O.E. (2021) High-resolution dietary reconstruction of victims of the 79 CE Vesuvius eruption at Herculaneum by compound-specific isotope analysis. *Science Advances,* **7,** eabg5791.
